# Dispensability of zinc and the putative zinc-binding domain in bacterial glutamyl-tRNA synthetase

**DOI:** 10.1042/BSR20150005

**Published:** 2015-03-31

**Authors:** Nipa Chongdar, Saumya Dasgupta, Ajit Bikram Datta, Gautam Basu

**Affiliations:** *Department of Biophysics, Bose Institute, P-1/12 CIT Scheme VIIM, Kolkata 700054, India; †Department of Biochemistry, Bose Institute, P-1/12 CIT Scheme VIIM, Kolkata 700054, India

**Keywords:** aminoacyl-tRNA synthetase, glutamine amidotransferase B (gatB), glutamyl-tRNA synthetase (GluRS), zinc-binding domain, zinc-binding motif, whole genome analysis, *Bb*-GluRS, *B. burgdorferri* GluRS, *Ec*-GluRS, *E. coli* GluRS, GatCAB, glutamine amidotransferase CAB, GlnRS, glutaminyl-tRNA synthetase, Glu-Q-RS, glutamyl-queuosine-tRNA^Asp^ synthetase, GluRS, glutamyl-tRNA synthetase, MMTS, methyl methanethiolsulfonate, *Mt*-GluRS, *M. tuberculosis* GluRS, PAR, 4-(2-pyridylazo) resorcinol, *pZBD*, putative zinc-binding domain, *Te*-GluRS, *T. elongatus* GluRS, *Tm*-GluRS1, *T. maritima* GluRS1, ZB-motif, zinc-binding motif

## Abstract

The putative zinc-binding domain (*pZBD*) in *Escherichia coli* glutamyl-tRNA synthetase (GluRS) is known to correctly position the tRNA acceptor arm and modulate the amino acid-binding site. However, its functional role in other bacterial species is not clear since many bacterial GluRSs lack a zinc-binding motif in the *pZBD*. From experimental studies on *pZBD*-swapped *E. coli* GluRS, with *Thermosynechoccus elongatus* GluRS, *Burkholderia thailandensis* GluRS and *E. coli* glutamyl-queuosine-tRNA^Asp^ synthetase (Glu-Q-RS), we show that *E. coli* GluRS, containing the zinc-free *pZBD* of *B. thailandensis*, is as functional as the zinc-bound wild-type *E. coli* GluRS, whereas the other constructs, all zinc-bound, show impaired function. A *pZBD*-tinkered version of *E. coli* GluRS that still retained Zn-binding capacity, also showed reduced activity. This suggests that zinc is not essential for the *pZBD* to be functional. From extensive structural and sequence analyses from whole genome database of bacterial GluRS, we further show that in addition to many bacterial GluRS lacking a zinc-binding motif, the *pZBD* is actually deleted in some bacteria, all containing either glutaminyl-tRNA synthetase (GlnRS) or a second copy of GluRS (GluRS2). Correlation between the absence of *pZBD* and the occurrence of glutamine amidotransferase CAB (GatCAB) in the genome suggests that the primordial role of the *pZBD* was to facilitate transamidation of misacylated Glu-tRNA^Gln^ via interaction with GatCAB, whereas its role in tRNA^Glu^ interaction may be a consequence of the presence of *pZBD*.

## Abstract 2

Zinc is functionally important in glutamylation of tRNA^Glu^ in *Escherichia coli*, yet, it is absent from many bacterial glutamyl-tRNA synthetases (GluRSs). We demonstrate and rationalize why zinc or the putative zinc-binding domain (*pZBD*) is not indispensable in all bacterial GluRSs.

## INTRODUCTION

Zinc plays an important structural and functional role in a number of aminoacyl-tRNA synthetases (aaRSs). For example, zinc is intimately related with cognate ligand recognition in *Escherichia coli* threonyl-tRNA synthetase [1] and cysteinyl-tRNA synthetase [2,3]. Similarly, a retrovirus like zinc-coordinating motif is important in tRNA recognition in *E. coli* alanyl-tRNA synthetase [4]. Chemical or mutational modification of the zinc-coordinating ligands in isoleucyl-tRNA synthetase (of *E. coli* and *Thermus thermophlilus*) affects the enzyme efficiency [5,6]. The crystal structure of *Methanosarcina bakeri* seryl-tRNA synthetase contains a Zn^2+^ ion in its active site; mutations of the zinc-coordinating ligands lead to the inactivation of the enzyme [7]. Removal of Zn^2+^ from *T. thermophlilus* methionyl-tRNA synthetase from many bacteria results in substantial loss of activity of the enzyme, although the zinc-binding motif (ZB-motif) lies far away from the active site [8].

**Figure 1:**
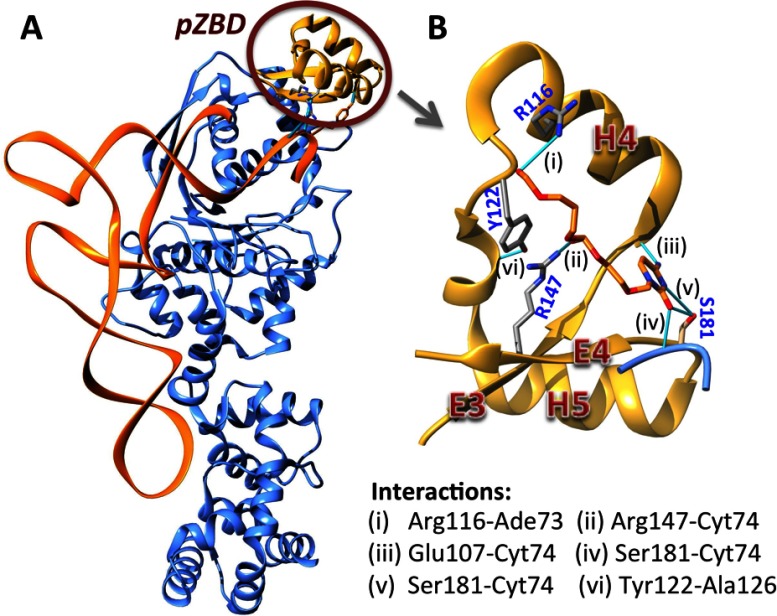
The *pZBD* in *T. thermophilus* GluRS and its interaction with tRNA^Glu^ (**A**) tRNA^Glu^-bound structure of *T. thermophilus* GluRS (pdb ID: 2dxi). (**B**) The *pZBD* (residues 101–150), defined by two helices H4 and H5 and capped by two β-strands E3 and E4 [orientations of *pZBD* in panels (**A** and **B**) are different for clarity]. Key H-bond interactions are highlighted: between tRNA^Glu^ (Ade^73^ and Cyt^74^) and *pZBD* residues (i, ii and iii), between tRNA^Glu^ and *pZBD* Ser^181^ (iv and v) and between Tyr^122^ and Ala^126^ (vi).

The presence of a Zn^2+^ in glutamyl-tRNA synthetase (GluRS) was first identified in the bacterium *E. coli* (*Ec*-GluRS) by X-ray fluorescence spectroscopy [9]. The removal of GluRS-bound Zn^2+^ reduced enzyme activity accompanied by a conformational change. Depletion of Zn^2+^ was inhibited in the presence of ATP, suggesting a close relationship between Zn^2+^ and the ATP-binding pocket. Partial proteolysis of *Ec*-GluRS followed by atomic absorption spectroscopic studies revealed the presence of Zn^2+^ in the N-terminal region of *Ec*-GluRS [9]. Extensive proteolytic analyses also showed that the ZB-motif is present in the fragment 98–138 of *Ec*-GluRS whereas Extended X-Ray Absorption Fine Structure data indicated that the co-ordination sphere of Zn^2+^ in *Ec-*GluRS consists of three sulfur and one nitrogen atoms [10]. Based on cysteine and histidine mutants of *Ec*-GluRS, present in the residue stretch 98-138, it was suggested that residues C98, C100, C125 and H127 coordinate Zn^2+^ ion in *Ec*-GluRS [10]. The *pZBD* of *Ec-*GluRS, containing the ZB-motif ^98^CxCx_24_CxH, belongs to the SWIM domain family that binds to DNA or protein [11,12]. An earlier study showed that the C100Y mutant of *Ec*-GluRS exhibits decreased glutamylation efficiency along with a reduced L-glutamic acid binding in presence of tRNA^Glu^ (L-glutamic acid binding remained unaffected in absence of tRNA^Glu^) without causing significant changes in the protein structure [12]. Close interaction of the putative zinc-binding domain (*pZBD*) with tRNA^Glu^ is also reflected in the tRNA^Glu^-bound structure of *T. thermophilus* GluRS (*Tt*-GluRS; Figure 1) [13].

Despite demonstrated structural and functional importance of the bound Zn^2+^ ion in *Ec*-GluRS, sequence analysis on a small set of bacterial GluRS showed that the putative ZB-motif CxCx_24_CxH (identified in *E. coli* GluRS) is absent from many bacteria, especially the third and fourth co-ordinating ligands [12]. This is corroborated from known crystal structures of bacterial GluRS, where Zn^2+^ is present in only one, GluRS from *Borrelia burgdorferri* (*Bb*-GluRS), out of six available crystal structures (Table 1), is zinc-bound. The role played by Zn^2+^ ion in bacterial GluRS therefore is poorly understood. Although it was proposed that the *pZBD* in different bacterial GluRS might have different local conformation and affinity for metal ions other than zinc [12], the proposal has not been examined in detail. We have experimentally studied a number of *Ec*-GluRS *pZBD*-chimeric constructs, with and without a ZB-motif, to understand the functional role of Zn^2+^. In addition, as part of our ongoing work on sequence analysis of a large database of bacterial GluRS from whole genome sequences [14], we have examined the occurrence of *pZBD* and ZB-motifs in GluRS across different bacterial phyla in conjunction with analyses of available GluRS structures. We show that a number of extant bacterial GluRS lack a *pZBD*. Our results imply a broader understanding of the functional role of the *pZBD* with or without bound Zn^2+^.

**Table 1:** GluRS and Glu-Q-RS structures in protein data bank

Protein	Organism	PDB ID	Status of zinc	zinc-binding motif
Bacterial GluRS	*B. burgdorferri*	4gri	Present	CXCX_20_YX_3_C
	*T. thermophilus*	2cuz, 2cvo, 2cv1, 2cv2, 1j09, 1n75, 1n77, 1n78, 2dxi	Absent	—
	*T. elongatus*	2cfo	Absent	CXCX_20_YX_3_H
	*M. tuberculosis*	2ja2	Absent	—
	*B. thailandensis*	4g6z	Absent	—
Bacterial GluRS1	*T. maritima* (TM1351)	3afh	Absent	—
Bacterial GluRS2	*T. maritima* (TM1875)	2o5r	Absent	—
Archaeal GluRS	*M. thermautotrophicus*	3aii	Present	CXCX_14_CXC
Bacterial Glu-Q-RS	*E. coli*	1nzj, 4a91	Present	CXCX_11_YX_3_C

## EXPERIMENTAL

### Cloning and purification of *pZBD*-chimeras of *Ec*-GluRS

A previously reported [15] *Ec*-GluRS encoding plasmid was used for the construction of *pZBD*-chimeras. Four sets of oligonucleotides were designed and were purchased from Integrated DNA Technologies. Using the above *Ec*-GluRS plasmid as a template, two separate sets of PCR reactions were performed with appropriate oligonucleotide combinations. The overall construction scheme along with the adequate oligonucleotide sequences is shown in Supplementary Figure S1. The resultant PCR products were cloned separately in the pETSUMO2 vector, as described previously [15]. All constructs were confirmed by DNA-sequencing (Applied Biosystems) and purified as described previously [15].

### Overproduction and purification of *E. coli* tRNA^Glu^

*E. coli* tRNA^Glu^ was overproduced *in vivo* using plasmid pKR15 (kindly provided by Professor Jacques Lapointe, Université Laval, Québec, Canada). *E. coli* DH5α containing pKR15 was grown at 37°C for ∼16 h in Luria broth media. The cells were harvested by centrifugation at 2700 ***g*** for 15 min and re-suspended in buffer containing 50 mM sodium acetate, 10 mM MgCl_2_ and 0.1 mM EDTA (pH 6). The RNA was then extracted in the aqueous layer using phenol–chloroform procedure (phenol was buffered with Tris/HCl, pH 7). The RNA was precipitated from the aqueous layer by using excess of pre-chilled isopropyl alcohol followed by incubation at −20°C for ∼20 h. The resulting white precipitate was separated by centrifugation at 16000 ***g*** at 4°C. The precipitate was dissolved in a buffer containing 20 mM HEPES (pH 7), 10 mM MgCl_2_, 100 mM NaCl and was loaded on to a 5 ml of HiTrap Q HP column (GE-healthcare). The column was washed with a buffer containing 20 mM HEPES, pH 7, 10 mM MgCl_2_ and 200 mM NaCl. Finally, purified tRNA^Glu^ was eluted with a NaCl gradient (300 mM, 1 M) in a buffer containing 20 mM HEPES, pH 7 and 10 mM MgCl_2_. Fractions containing purified tRNA were confirmed by 10% urea/PAGE and were extensively dialysed in sterile water. The purified tRNA samples were then lyophilized and dissolved in diethylpyrocarbonate treated Mili-Q water. The acceptor activity of the purified tRNA^Glu^ was measured to be ∼1.1 nmol/*D*_260_.

### Structural studies by CD and fluorescence spectroscopy

Far-UV CD (200—260 nm) studies were performed using a Jasco J-815 spectro-polarimeter at 25°C in 50 mM phosphate buffer (pH 7.5) containing 100 mM NaCl using a cuvette of 2-mm path-length and a protein concentration of 10 μM. The Near-UV CD spectra (260—330 nm) of the protein samples (∼10 μM) were recorded in the same machine but with a 10-mm path-length cuvette. Steady state fluorescence spectra (310–400 nm) of the protein samples (∼5 μM each) were recorded in a Hitachi F7000 spectrofluorimeter, using a cuvette of path-length of 5 mm (excitation wavelength: 295 nm). All of the above experiments were carried out at 25°C. Protein concentrations were determined spectrophotometrically (absorption at 280 nm) for this and all following studies.

### Binding affinities of tRNA^Glu^ and ATP using fluorescence spectroscopy

Binding of substrates (tRNA^Glu^ and ATP) with *Ec-*GluRS and the *pZBD*-chimeras were monitored by tryptophan-fluorescence quenching experiments in a Hitachi F7000 spectro-fluorimeter. All binding experiments were performed in 20 mM HEPES buffer, pH 7.5, containing 5 mM MgCl_2_, with an enzyme concentration of 0.5 μM (for *Ec-*tRNA^Glu^ titrations) and 2 μM (for ATP titrations) at 25°C. Binding of *Ec-*tRNA^Glu^ was monitored by single point titration as described before [16]. The excitation and emission wavelengths were 295 and 340 nm respectively. The resulting binding isotherms were analysed using standard equations assuming a 1:1 binding stoichiometry, as described earlier [17].

### Glutamylation assay

For the glutamylation assay, uniformly labelled [3,4-^3^H] L-glutamic acid was purchased from Perkin–Elmer, with a specific radioactivity is 50 Ci/mmol. *In vitro* glutamylation assay of the wild-type and the *pZBD*-chimeras of *Ec-*GluRS were carried out with 4 μM of the previously isolated *Ec*-tRNA^Glu^ in 50 mM HEPES (pH 7.5), 0.1 mM unlabelled L-glutamic acid, 16 mM MgCl_2_, 2 mM ATP, 0.8 mM β-mercaptoethanol and [3,4-^3^H] L-Glu (0.5 μl of stock per 100 μl of assay buffer) at 37°C using a methodology described earlier [16]. Kinetic parameters (*K*_m_ and *k*_cat_) associated with the glutamylation reactions were determined with respect to L-glutamic acid (50-300 μM) at 37°C as described earlier [18].

### Measurement of zinc-content

The zinc-contents of *Ec*-GluRS and *pZBD*-chimeras were measured using a spectroscopic method [19] with methyl methanethiolsulfonate (MMTS) as the cysteine modifier [20] and 4-(2-pyridylazo) resorcinol (PAR) as the zinc-sensitive probe that displays ε_max_ at 416 and 500 nm in zinc-free and zinc-bound states respectively. Aliquots of 10 μM proteins were incubated with 50 μM PAR in 50 mM Tris/HCl (pH 7) buffer containing 500 mM NaCl and the absorbances at 500 nm were recorded (as background). Protein-bound zinc was released by adding 100 μM MMTS (from DMSO stock solution) and the time course of Zn–(PAR)_2_ formation was monitored by measuring the absorbance at 500 nm as a function of time for 10 min. Concentrations of protein-bound zinc were estimated by correlating the background-subtracted absorbance at 500 nm with a standard curve where the protein in the above protocol was replaced by known amounts of ZnSO_4_. All experiments were carried out at 25°C.

### Database construction and sequence analysis

A total of 212 bacterial GluRS sequences (Supplementary Figure S2; Supplementary Table S1) were analysed from a database published earlier [14]. In addition, 61 bacterial Glu-Q-RS sequences (Supplementary Figure S3; Supplementary Table S2), 31 archaeal GluRS sequences (Supplementary Figure S4; Supplementary Table S3), 22 eukaryal GluRS and 11 glutamyl-prolyl-tRNA synthetase (GluProRS) sequences (Supplementary Figure S5; Supplementary Table S4) were compiled from Kyoto Encyclopedia of Genes and Genomes database [21]. Using a methodology published earlier, sequences were aligned using PROMALS3D [22], with default parameters using X-ray structures of bacterial GluRS, archaeal GluRS and bacterial Glu-Q-RS. Structural alignments were performed using by MATRAS [23] with default parameters.

## RESULTS

### Analysis of *pZBD*s and ZB-motifs in bacterial GluRS

**Figure 2:**
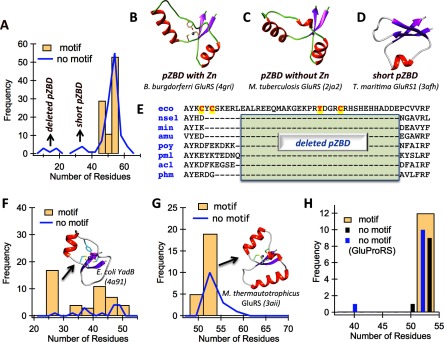
Analysis of *pZBD* in GluRS and Glu-Q-RS (**A**) Length distributions of *pZBD*s in bacterial GluRS and representative structures of canonical length bacterial *pZBD* with zinc. (**B**) A canonical length *pZBD* with zinc. (**C**) A canonical length *pZBD* without zinc. (**D**) A short *pZBD* without zinc. (**E**) *pZBD*-deleted bacterial GluRSs (eco: *E. coli*; nse1: *N. sennetsu* GluRS1; min: *M. infernorum*; amu: *A. muciniphila*; poy: *Onion yellows phytoplasma*; pml: *P. mali*; acl: *A. laidlawii*; phm: *P. mikurensis*). (**F**) Length distributions of *pZBD*s in bacterial Glu-Q-RS. (**G**) Length distributions of *pZBD*s in archaeal GluRS. (**H**) Length distributions of *pZBD*s in eukaryal GluRS/GluProRS.

Among the six available structures *pZBD*s present in bacterial GluRS (Table 1) only *Bb*-GluRS is zinc-bound, chelated by the CxCx_20–21_Yx_3_C motif (bold letters indicate co-ordinating residues). *Ec*-GluRS, for which only a preliminary crystallization report is available [15], also displays an identical ZB-motif CxCx_20_Yx_3_C (the histidine mentioned in the introduction section appears after the motif as: CxCx_20_Yx_3_CxH). The bound Zn^2+^ in *Ec-*GluRS was shown to be functionally important [9,10,12]. Yet, *pZBD*s in many bacterial GluRS sequences lack a characteristic ZB-motif [12]. We investigated the presence (or absence) of ZB-motifs and the length distribution of *pZBD*s in a database of 212 bacterial GluRS sequences (Table 2; Figure 2; Supplementary Figure S2). A ZB-motif was present in about 44% (93) cases in the database. As shown in Figure 2(A), the length of *pZBD*s mostly varied between 46 and 54 residues. However, for *pZBD*s without a ZB-motif, two minor populations, with significantly shorter lengths (seven each in the range 31–36 and 10–17), were also observed.

**Table 2:** A Summary of ZB-motifs in GluRS and Glu-Q-RS

	Protein (total)	Group (total)	ZB-motif
Bacteria	GluRS (212)	I (88)	CxCx_20–21_Yx_3_C
		II (5)	CxCx_20_Yx_3_H
		III (105)	No ZB-motif (long *pZBD*)
		IV (7)	No ZB-motif (short *pZBD*)
		V (7)	*pZBD-*deleted
	Glu-Q-RS (61)	I (46)	CxCx_11–28_Yx_3_C
		II (15)	No ZB-motif
Archaea	GluRS (37)	I (7)	CxCx_14_CxC
		II (16)	CxCx_14_CxH
		III (14)	No ZB-motif
Eukarya	GluRS (22)	I (11)	CxCx_20_Yx_3_C
		II (1)	CxCx_20_Cx_3_C
		III (10)	No ZB-motif
	GluProRS (11)	11	No ZB-motif

We classified bacterial GluRSs into five groups (Table 2) based on a multiple sequence alignment of the entire database (Supplementary Figure S2). The first group (88 GluRS) contained the canonical ZB-motif CxCx_20–21_Yx_3_C. The second group (five GluRS) contained a modified ZB-motif CxCx_20_Yx_3_H. The third and the largest group (105 GluRS) did not display any discernable ZB-motif. Available crystal structures [24–26] show that the core architecture of *pZBD*s remains unaltered with (Figure 2B) or without a bound Zn^2+^ ion (Figures 2B and 2C). The seven GluRS sequences, with length 31–36 (marked as ‘short *pZBD*’ in Figure 2A), form the outlier fourth group. The crystal structure of one *pZBD* in this category from *Thermatoga maritima* GluRS1 (*Tm*-GluRS1) [26] shows that the short *pZBD* can adopt a non-canonical folded structure (Figure 2D). Surprisingly, all members of the fifth group (seven with length 10–17) had their *pZBD*s deleted (marked as ‘deleted *pZBD*’ in Figure 2A), except for the first and the last (*E3* and *E4*; see Figure 1B) β-strands (see Figure 2E for *pZBD* sequence alignment).

### *pZBD*s and ZB-motifs in bacterial Glu-Q-RS and GluRS from archaea and eukarya

For comparison, we also analysed representative *pZBD*s from Glu-Q-RS (Supplementary Figure S3), a catalytic-domain-only paralogue of bacterial GluRS [27]. The crystal structure of *E. coli* Glu-Q-RS [27,28], is also bound to a Zn^2+^ ion (inset to Figure 2F), ligated by three cysteine and one tyrosine residues and present in the motif CxCx_11–28_Yx_3_C. This motif is present in 46 (out of 61) Glu-Q-RS sequences. The length distribution of *pZBD*s with a ZB-motif showed a broad distribution around 45 with a sharp peak at 25 (corresponding to *E. coli* Glu-Q-RS); *pZBD*s without a ZB-motif also show a broad distribution.

The N-terminal domain of GluRS containing the *pZBD* is thought to be of ancient origin and is homologous in bacteria, archaea, as well as in eukaryotes [29]. We analysed *pZBD*s from representative archaea and eukaryotic GluRSs. The ZB-motif in archaeal GluRS, CxCx_14_CxH or CxCx_14_CxC (present in *Methanothermobacter thermautotrophicus* GluRS whose structure [30] is shown in the inset to Figure 2G), is slightly different from that present in bacterial GluRS. The length distributions of archaeal *pZBD*, with (23 out of 37) or without a ZB-motif, are very similar to each other, with a peak at 53 (Figure 2G). Out of a total of 33 eukaryal GluRS sequences examined here (including 11 GluProRS), 11 show the bacteria-like ZB-motif CxCx_20_Yx_3_C, whereas one showed the archaeal motif CxCx_20_Cx_3_C. The lengths of all *pZBD*s are restricted mostly to 54 residues (Figure 2H).

### Phylum-specific distribution of *pZBD* in bacterial GluRS

**Figure 3:**
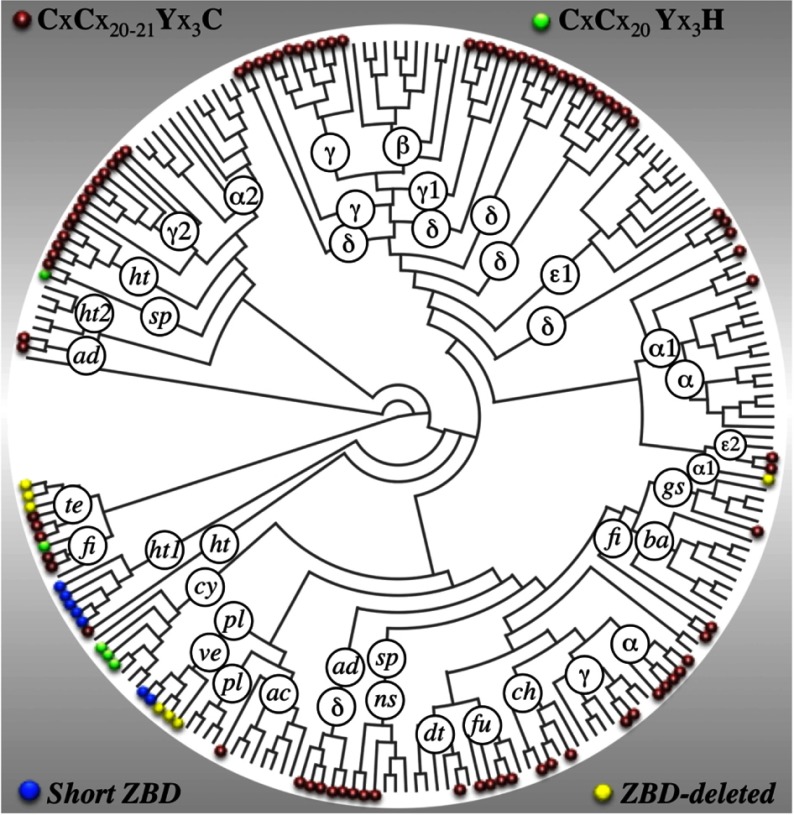
Phylogenetic tree of bacterial GluRS with and without ZB-motifs Projection of ZB-motifs (present in *pZBD*s) on to the phylogenetic tree of bacterial GluRS. The abbreviations stand for bacterial phyla and classes: proteobacteria (α, β, δ, ε, γ), hyperthermophilic bacteria (ht), acidobacteria (ad), spirochaetes (sp), bacteroidetes (ba), chlamydiae (ch), fusobacteria (fu), deinococcus-thermus (dt), (ns), green sulfur bacteria (gs), planctomycetes (pl), verrucomicrobia (ve), cyanobacteria (cy), actinobacteria (ac), firmicutes (fi), tenericutes (te). Suffixes 1 and 2 stand for GluRS1 and GluRS2 respectively (no suffix signifies canonical GluRS).

The appearance of myriad *pZBD*s in bacterial GluRS calls for their origin and evolutionary history. We addressed this by projecting the ZB-motifs on to a previously published phylogenetic tree of the parent GluRS sequences [14]. The ZB-motif-annotated GluRS phylogenetic tree shows that specific motifs mostly appear in monophyletic clades with a phylum-specific preference (Figure 3). The canonical motif (CxCx_20–21_Yx_3_C; group-I Table 2) is associated with specific proteobacterial classes like γ (GluRS, GluRS1 and GluRS2; where GluRS1 and GluRS2 refer to two copies of non-identical GluRS present in some bacteria [31] where GluRS1 is more specific to tRNA^Glu^ whereas GluRS2 is specific to tRNA^Gln^), δ (GluRS) and α (especially GluRS that cluster with non-proteobacteria) and non-proteobacterial phyla like fusobacteria (GluRS), firmicutes (GluRS), hyperthermophilic bacteria (GluRS). The canonical motif also appears in spirochetes, green non-sulfur bacteria (GluRS), tenericutes, chlamydae and acidobacteria, although not in a strict sense. A slightly altered motif (CxCx_20_Yx_3_H; group-II, Table 2) occurs in three cyanobacteria (GluRS), one spirochaete (GluRS) and one firmicute (GluRS). Seven GluRSs with short *pZBD* (group-IV, Table 2) appear mostly as GluRS1 in hyperthermophilic bacteria in addition to two occurrences in verrucomicrobia (GluRS). The seven *pZBD*-deleted GluRSs sequences (group-V, Table 2; Figure 2E), three tenericutes (GluRS), two verrucomicrobia (GluRS), one plancomycets (GluRS) and one α-proteobacterim (GluRS1), mostly form monophyletic groups.

### Superposition of *pZBD*s of known GluRS structures with and without a ZB-motif

**Figure 4:**
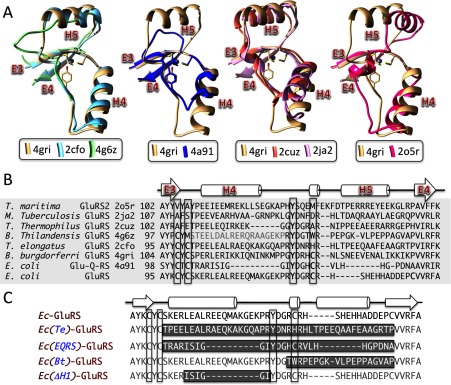
Superposition of GluRS *pZBD*s and the design of *Ec*-GluRS *pZBD-*chimeras (**A**) Structural superposition and (**B**) structure-based multiple sequence alignment of *pZBD*s from bacterial GluRS with known crystal structures (Table 1). Structural superposition as shown here was restricted to the first and the last β-strands only (E3 and E4). RMSD and the number of superposed atoms for *pZBD* from 2o5r, 2ja2, 2cuz, 4a91, 2cfo and 4g6z (against *pZBD* of 4gri) were 0.804 Å (22), 0.884 Å (37), 0.939 Å (32), 0.333 Å (23), 0.805 Å (54) and 0.576 Å (16) respectively. (**C**) Sequence of *pZBD*s of *Ec*-GluRS and four *pZBD*-chimeras used in this work. The grey-highlighted sequence stretches were grafted into the corresponding sequence blocks in *Ec*-GluRS *pZBD* in the chimeras. The four zinc-binding residues are highlighted in panels (**B**) and (**C**).

The absence of ZB-motif in about ∼ 60% cases suggests that the role of Zn^2+^ is not universally important in bacterial GluRS. By co-ordinating four non-contiguous amino acids, the bound Zn^2+^ is expected to play a structural role in ZB-motif-containing GluRS. What about structures lacking a bound Zn^2+^ despite displaying a ZB-motif [25] or those with a disrupted ZB-motif? Superposition of the *pZBD*s from known GluRS structures (Figures 4A and 4B) showed that the core fold, defined by two consecutive helices (H4 and H5) capped by two β-strands (E3 and E4), is maintained in almost all structures, with or without zinc. Minor exceptions are: (i) *E. coli* Glu-Q-RS [27,28] where the first helix H4 is short (two-turn) and the second helix H5 is lost, (ii) *Burkholderia thailandensis* GluRS (*Bt*-GluRS) [32] where the first helix H4 is disordered in the crystal structure and the second helix H5 is missing, (iii) *Mycobacterium tuberculosis* GluRS (*Mt*-GluRS) [33] and *Tt*-GluRS [34] where the first helix H4 is short (three-turn) with a one-turn helix between the two longer helices H4 and H5 and, (iv) *T. maritima* GluRS2 (*Tm*-GluRS2) [26] which also displays a one-turn helix between the two longer helices H4 and H5, the latter displaced compared with *Bb*-GluRS. The overall conservation of the core fold suggests that bacterial GluRS without Zn^2+^ may exploit an alternative strategy to maintain the local structural scaffold.

### Designing the *pZBD*-chimeras of *Ec*-GluRS

To understand the role played by Zn^2+^ in bacterial GluRS, we focused on *Ec*-GluRS and constructed four chimeric versions (Figure 4C) where part of the *Ec*-GluRS *pZBD* was replaced by a stretch of residues from other bacterial GluRS *pZBD*s. The *pZBD* can best be defined from a structural superposition of known GluRS structures as the domain anchored by a two-stranded (E3 and E4) β-sheets (Figures 1B and 4). In the first chimera [*Ec(Te)-*GluRS], the *pZBD* of *Ec*-GluRS was replaced by the corresponding *pZBD* from *Thermosynechococcus elongatus* GluRS (*Te*-GluRS) whose structure [25] is devoid of a bound Zn^2+^ despite containing the modified ZB-motif CxCx_n_Yx_3_H. In the second chimera [*Ec(EQRS)*-GluRS] the *pZBD* of *Ec*-GluRS was replaced by the corresponding *pZBD* from *E. coli* Glu-Q-RS (*Ec*-EQRS) whose zinc-bound structure [28] contains the ZB-motif CxCx_n_Yx_3_C. In the third chimera [*Ec(Bt)*-GluRS], the *pZBD* of *Ec*-GluRS was replaced by a 19-residue stretch from the *pZBD* of *Bt*-GluRS which contains a disrupted ZB-motif CxMx_20_Yx_3_W and whose structure is devoid of a bound Zn^2+^ ion [32]. The 19-residue stretch starts from the residue preceding the fourth zinc-co-ordinating cysteine residue in *Ec*-GluRS and continues until the last β-strand of the *pZBD* fold. In most other GluRS (Figure 4B), this stretch adopts a helical structure but not in *Bt*-GluRS. It is also this stretch where the *pZBD*s of *Ec*-GluRS and *Bt*-GluRS differ the most. In addition, a *pZBD*-disrupted *Ec*-GluRS was constructed [*Ec(ΔH4)*-GluRS] where the ZB-motif of *Ec*-GluRS was left untouched but the first helix was shortened by grafting the corresponding stretch from *Ec*-EQRS in which the first helix is only two-turn long. All four variants of *Ec*-GluRS, along with the wild-type enzyme, were overexpressed and purified for structural and functional studies.

### The structural integrity of *Ec*-GluRS remains unaltered by *pZBD* perturbations

Far-UV CD spectroscopy is an important tool to study secondary structure of the polypeptide whereas near-UV CD spectroscopy is a tool to gauge tertiary structure of proteins. Intrinsic protein fluorescence is a complementary tool that can report the solvent accessibility tryptophan residues in proteins. To identify the overall structural perturbation in *pZBD*-chimeras CD and fluorescence spectroscopy were performed and compared with that of *Ec*-GluRS.

**Figure 5:**
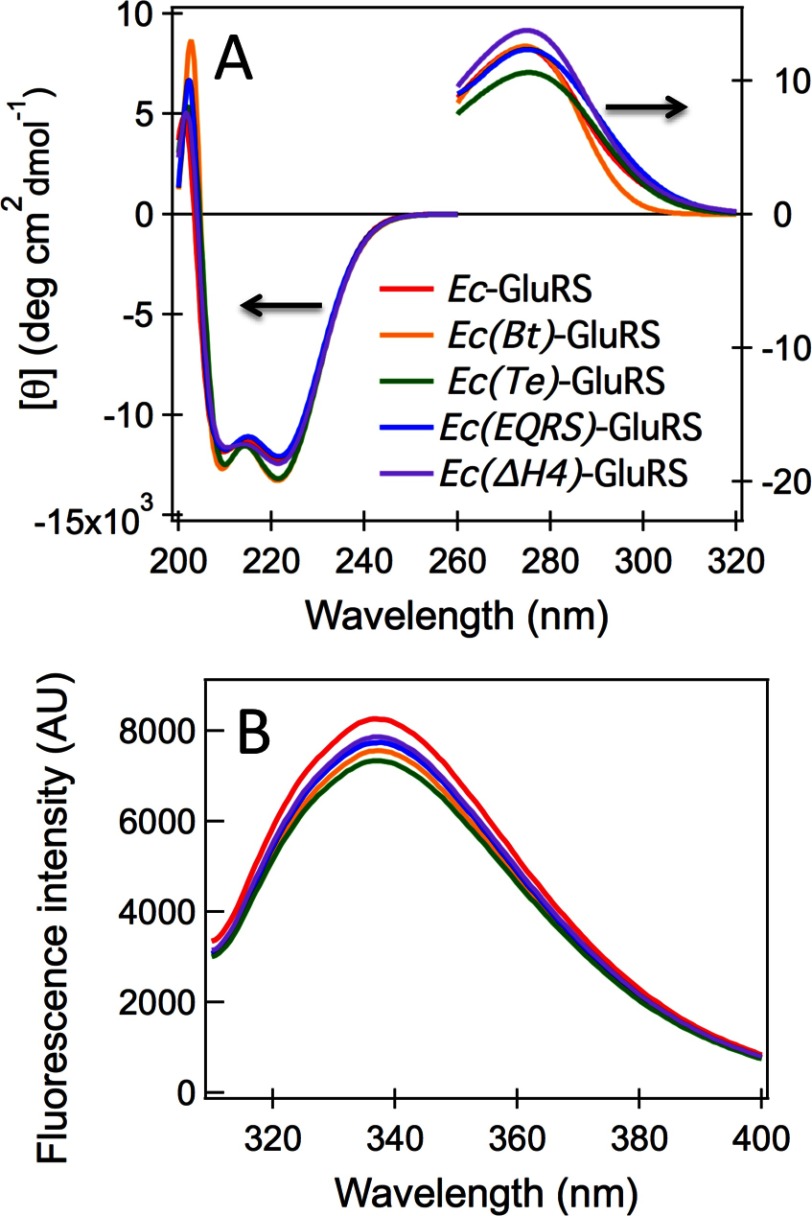
Solution conformational studies of *Ec*-GluRS and *pZBD*-chimeras (**A**) CD spectra of *Ec*-GluRS variants in phosphate buffer (pH 7.5) containing 100 mM NaCl at 25°C. (**B**) Fluorescence spectra of *Ec*-GluRS variants under identical conditions as panel (**A**).

The far-UV CD spectra of wild-type *Ec*-GluRS, *Ec(Bt)*-GluRS, *Ec(Te)*-GluRS, *Ec(EQRS)*-GluRS and *Ec(ΔH4)*-GluRS are comparable (Figure 5A), an indication of very similar secondary structure contents. The fact that secondary structural content of *Ec*-GluRS is comparable to that of the *pZBD*-chimeras with shorter *pZBD*s [*Ec(EQRS)*-GluRS and *Ec(ΔH4)*-GluRS] possibly indicates that the longer stretch of *pZBD* in *Ec*-GluRS, not present in *Ec(EQRS)*-GluRS or *Ec(ΔH4)*-GluRS, may actually be devoid of canonical secondary structure. In summary, the secondary structures of all three *pZBD*-chimeras of *Ec*-GluRS are similar to that of wild-type *Ec*-GluRS. The near-UV CD spectra of *Ec*-GluRS and the *pZBD*-chimeras are shown in Figure 5(A). The nature and intensity of the near-UV CD spectra are also comparable to the wild-type protein, indicating that the tertiary structure of *Ec*-GluRS remains almost unaltered after *pZBD*-swapping.

Apart from *Bt*-ZBD, which has nine tryptophan residues, all *pZBD*s in the chimeras and in *Ec*-GluRS have eight tryptophan residues each. The emission maxima in tryptophan fluorescence spectra of all chimeras and wild-type *Ec*-GluRS were identical (*λ*_max_=334 nm; Figure 5B). The relative intensities were also comparable indicating that that replacement *pZBD* of *Ec*-GluRS does not affect the overall solvent exposure of tryptophan residues. Therefore, it can be concluded from the spectroscopic analysis that the overall secondary structure and compactness of wild-type *Ec*-GluRS remains unaltered in the chimeric constructs studied in the present paper.

### Association of GluRS with tRNA^Glu^ but not with ATP is sensitive to *pZBD* perturbations

Efficient glutamylation demands optimal binding of substrates (ATP, tRNA^Glu^ and L-glutamic acid) by GluRS. Binding of tRNA^Glu^ induces conformational changes in GluRS that stimulates the binding of L-glutamic acid leading to the productive binding of ATP [16,34,35]. Substrate binding is known to quench tryptophan fluorescence [17]. Therefore, the extent of substrate binding to *Ec*-GluRS can be monitored by fluorescence titration experiments. The binding affinities of ATP and *Ec*-tRNA^Glu^ towards *pZBD*-chimeras of *Ec*-GluRS were measured from tryptophan fluorescence detected titrations and compared with that of wild-type *Ec*-GluRS.

It was shown by Liu et al. [9] that 1,10-phenanthroline-mediated zinc depletion and inactivation of *Ec*-GluRS is inhibited in the presence of ATP. Even at an elevated temperature of 50°C ATP strongly hindered the depletion of zinc, indicating a close association between *pZBD* and the ATP-binding pocket [9]. Therefore it is instructive to demonstrate how *pZBD*-swapped versions of *Ec*-GluRS bind ATP. The dissociation constants of ATP (*K*_d_), derived from tryptophan fluorescence titration (Table 3), shows that affinity of ATP to *Ec*-GluRS (28.0 μM) is comparable to its *pZBD*-variants [*Ec(Bt)*-GluRS: 39.5 μM; *Ec(Te)*-GluRS: 32.0 μM; *Ec(EQRS)*-GluRS: 35.5 μM; *Ec(ΔH4)*-GluRS: 28.0 μM]. Thus, swapping of *pZBD* of *Ec*-GluRS by *pZBD*s from other GluRSs or even with the *pZBD* of *Ec*-Glu-Q-RS does not alter the ATP-binding activity.

**Table 3:** Kinetic assay parameters and zinc-content of *Ec*-GluRS and its variants

*Ec-*GluRS *constructs*	*k*_cat_ (s^−1^)	*K_m_* (L-glutamic acid; 10^−6^ M)	*k*_cat_*/K*_m_ (L-glutamic acid; M^−1^·s^−1^)	Activity loss	*K*_d_ (ATP) (10^−6^ M)	*K*_d_ (tRNA^Glu^) (10^−9^ M)	[Zn^2+^]/[protein]* (molar ratio)
wt *Ec-*GluRS	5.3±0.1	65.4±6.7	8.1×10^4^	–	28.0±2.8	62.5±6.3	0.93±0.01
*Ec(Bt)-*GluRS	6.6±0.7	139.4±3.7	4.7×10^4^	∼2	39.5±0.7	91.0±7.0	0.05±0.01
*Ec(Te)-*GluRS	0.20±0.03	250.5±4.0	8.0×10^2^	∼100	32.0±4.2	222±5.6	1.08±0.08
*Ec(EQRS)-*GluRS	(1.5±0.2) × 10^−2^	284.6±3.0	53	∼1500	35.5±0.7	385±19	1.09±0.05
*Ec-(ΔH4)-*GluRS	(8.5±0.1) × 10^−3^	441.5±9.2	19	∼4200	28.0±1.4	499±8.4	1.00±0.05

*Under identical experimental conditions, [Zn^2+^]/[*Ec-*GlnRS]=0.06±0.01.

Abbreviation: wt, wild-type.

The *pZBD* of *Ec*-GluRS has been proposed to interact with the acceptor arm of *Ec*-tRNA^Glu^ and impart higher selectivity of L-glutamic acid over other non-cognate amino acids by assisting in reorganizing the active site of GluRS [12]. The fluorescence titration derived *K*_d_ values (Table 3) of *Ec*-tRNA^Glu^ dissociation from *Ec(Bt)*-GluRS (91.0 nM) and *Ec(Te)*-GluRS (222 nM) are about 1.4- and 3.5-times higher than that of wild-type *Ec*-GluRS (62.5 nM). This indicates that *pZBD* of *Te*-GluRS is not as compatible as the *pZBD* of *Bt*-GluRS towards *Ec-*tRNA^Glu^. The *K*_d_ values (Table 3) of *Ec*-tRNA^Glu^ dissociation from *Ec(EQRS)*-GluRS (385 nM) and *Ec(ΔH4)*-GluRS (499 nM) are ∼6- and 8-fold higher than that of *Ec*-GluRS. Compared with *Ec*-GluRS, the free energies of *Ec*-tRNA^Glu^-binding (*ΔG^0^=−RTlnK*_d_) to *Ec(Bt)*-GluRS, *Ec(Te)*-GluRS, *Ec(EQRS)*-GluRS and *Ec(ΔH4)*-GluRS are unfavourable by 0.22, 0.75, 1.07 and 1.23 kcal/mol (1 cal ≡ 4.184 J) respectively. Overall, the experiments showed that tRNA^Glu^ binding to GluRS is sensitive to *pZBD* perturbations.

### Except *Ec(Bt)*-GluRS all *pZBD*-chimeras show 100-fold or more weaker catalytic efficiency

Glutamylation of tRNA^Glu^ by GluRS is the exclusive pathway for Glu-tRNA^Glu^ synthesis. In this reaction, L-glutamic acid is first activated by GluRS in presence of ATP to form the adenylate complex. This is followed by the catalytic step where the acceptor stem of tRNA^Glu^ is glutamylated. In the light of the weaker affinities of tRNA^Glu^ for most *pZBD*-chimeras, one would expect poor glutamylation efficiencies. However, the effect of the *pZBD*-perturbations on the catalytic step (*k*_cat_) may not necessarily be reflected in the weaker *K*_d_ values. The kinetic parameters (*k*_cat_ and *K*_m_) for tRNA^Glu^ glutamylation of *Ec*-GluRS and *pZBD*-chimeras were measured by varying L-glutamic acid concentration.

The *K*_m_ variation of the *pZBD*-mutants showed a trend very similar to that of *K*_d_ (GluRS–tRNA^Glu^) variation. Compared with the *K*_m_ of wild-type *Ec*-GluRS (65.4 μM), the *K*_m_ values of the *pZBD*-chimeras (Table 3) were enhanced: *Ec(Bt)*-GluRS (2.1-fold), *Ec(Te)*-GluRS (3.8-fold), *Ec(EQRS)*-GluRS (4.3-fold) and *Ec(ΔH4)*-GluRS (6.7-fold). In terms of free energies, the association process is unfavourable by 0.45, 0.79, 0.87 and 1.13 kcal/mol respectively, for the chimeras. The *k*_cat_ variation of the *pZBD*-mutants (Table 3), on the other hand, showed a slightly different trend. Although *k*_cat_ values for *Ec*-GluRS (5.3 s^−1^) and *Ec(Bt)*-GluRS (6.6 s^−1^) were comparable, *k*_cat_ values of other chimeras were order of magnitude smaller: *Ec(Te)*-GluRS (26-fold), *Ec(EQRS)*-GluRS (353-fold) and *Ec(ΔH4)*-GluRS (623-fold). The overall efficiency of the glutamylation step is reflected in the ratio *k*_cat_/*K*_m_ (Table 3). Compared with *Ec*-GluRS (*k*_cat_/*K*_m_=8.1×10^4^ M^−1^ s^−1^), the *k*_cat_/*K*_m_ ratios for *Ec(Te)*-GluRS, *Ec(EQRS)*-GluRS and *Ec(ΔH4)*-GluRS were lower by ∼ 100-fold, 1500-fold and 4200-fold respectively. On the other hand, the *k*_cat_/*K*_m_ ratios for *Ec*-GluRS and *Ec(Bt)*-GluRS (*k*_cat_*/K*_m_=4.7×10^4^ M^−1^ s^−1^) were comparable.

In summary, the above experimental results suggest that the *pZBD* in GluRS not only affects the local interaction with tRNA^Glu^ acceptor arm (thus modulating the active site) but also affects the catalytic step of glutamylation reaction indicating some distant communication with other part of the protein. Among the three *pZBD*-variants, favourable long-range communication between this domain and the rest of the protein is most severely hampered in *Ec(EQRS)*-GluRS and *Ec(ΔH4)*-GluRS, both with a shorter H4 helix than *Ec*-GluRS (Figure 4C). This suggests that the H4 helix of *Ec*-GluRS *pZBD* is important for glutamylation of tRNA^Glu^. Interestingly tRNA^Glu^–H4 helix interaction was also observed in the crystal structure of *Tt*–GluRS–tRNA^Glu^ complex (Figure 1).

### Except *Ec(Bt)*-GluRS all *pZBD*-chimeric constructs contain zinc

**Figure 6:**
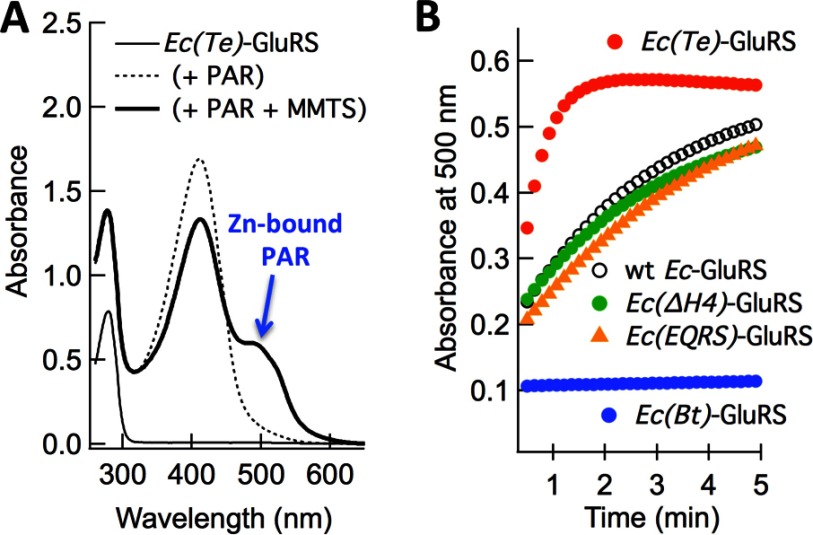
Zinc-content of *Ec*-GluRS and *pZBD*-chimeras (**A**) UV-vis spectra of pure *Ec(Te)*-GluRS (10 μM) and its mixture with PAR (50 μM) and MMTS (100 μM) in Tris/HCl buffer (pH 7) containing 500 mM NaCl at 25°C. (**B**) Time evolution of zinc-bound PAR (absorbance at 500 nm) upon addition of PAR (50 μM) to a mixture of MMTS (100 μM) and *Ec*-GluRS variants (∼10 μM) in Tris/HCl buffer (pH 7) containing 500 mM NaCl at 25°C.

The zinc-content of *Ec*-GluRS and all *pZBD* chimeras were spectroscopically determined from the absorbance of zinc-bound PAR at 500 nm (Figure 6A) [19]. The experimentally determined protein–zinc molar ratios are summarized in Table 3. Moles of zinc, released from each mole of protein, were 0.93 (*Ec*-GluRS), 1.09 [*Ec(EQRS)*-GluRS], 1.08 [*Ec(Te)*-GluRS] and 1.0 [*Ec(ΔH4)*-GluRS], confirming that the *pZBD*s of these proteins are zinc-bound. This is not unexpected since the presence of zinc in *Ec*-GluRS was reported earlier [9,10,12]. Similarly, the *pZBD* of *Ec(EQRS)*-GluRS is expected to bind zinc since EQRS–*pZBD* contains a ZB-motif and *Ec*-EQRS crystal structure also contains zinc [27,28] Likewise, the *pZBD* of *Ec(ΔH4)*-GluRS is almost identical with that in *Ec*-GluRS except that the first helix H4 in *pZBD* of *Ec(ΔH4)*-GluRS is shortened without disturbing the four potential zinc-co-ordinating residues. Therefore, that it also released one equivalent of zinc is not surprising. The crystal structure of *Te*-GluRS does not contain zinc despite having a modified ZB-motif [25] Therefore, the presence of zinc in *Ec(Te)*-GluRS was a little unexpected.

Although it contains one equivalent of zinc, the kinetics of zinc-release by *Ec(Te)*-GluRS was different from others (Figure 6B). It took *Ec*-GluRS, *Ec(EQRS)*-GluRS and *Ec(ΔH4)*-GluRS about 5 min to release about 90% of the bound zinc. In contrast, almost all the bound zinc in *Ec(Te)*-GluRS was released within a minute. The zinc-chelator MMTS reacts with cysteine thiol groups to release zinc [20], therefore the presence of only two cysteines in *Ec(Te)*-GluRS, as opposed to three in the rest [*Ec*-GluRS, *Ec(EQRS)*-GluRS and *Ec(ΔH4)*-GluRS] explains why zinc-release was much faster in *Ec(Te)*-GluRS. This also makes *Ec(Te)*-GluRS a weaker binder of zinc than the rest. Interestingly, *Bacillus subtilis* GluRS, with an identical ZB-motif as *Ec(Te)*-GluRS (CxCx_n_Yx_3_H) also binds to zinc but with a weak affinity as evident from the fact that only 0.6 moles of zinc (per mole of protein) was found to be protein-bound [9]. We presume that the apparently loosely bound zinc in *Ec(Te)*-GluRS was lost during the crystallization process due to the presence of mild chelators (like sodium citrate and citric acid) in the crystallization cocktail and as a result the crystal structure is devoid of any bound zinc.

In contrast with the above results, the chimera *Ec(Bt)*-GluRS released 0.05 moles of zinc per mole of protein. This number is comparable to the control (0.06 mole of zinc), *E. coli* glutaminyl-tRNA synthetase (GlnRS), which is known to not bind zinc [9]. That *Ec(Bt)*-GluRS does not bind zinc is expected since *Ec(Bt)*-GluRS contains a disrupted ZB-motif CxCx_20_Yx_3_W.

## DISCUSSION

### Natively zinc-bound *Ec*-GluRS does not require zinc to be active

The aim of the present study is to revisit the functional and structural role of the *pZBD*, present in the N-terminal catalytic domain of bacterial GluRS (Figure 1). Previous experimental work on *Ec*-GluRS, where the ZB-motif in *pZBD* was disrupted or the Zn^2+^ was removed, showed that the Zn^2+^ ion plays an important functional role [9,10]. We revisited this conclusion by constructing three chimeric versions of *Ec*-GluRS where *pZBD*s from *Te*-GluRS, *Bt*-GluRS and *Ec*-Glu-Q-RS replaced the *pZBD* of *Ec*-GluRS (Figure 4C). The fourth chimera had a *pZBD* with shortened H4 helix. Despite being zinc-bound, *Ec(Te)*-GluRS, *Ec(EQRS)*-GluRS and *Ec(ΔH4)*-GluRS showed impaired enzymatic efficiencies. On the other hand, *Ec(Bt)*-GluRS showed comparable enzymatic efficiency as the wild-type GluRS despite lacking the canonical ZB-motif or a bound Zn^2+^. The results clearly demonstrate that Zn^2+^ ion, by itself, is not required for the function of *Ec*-GluRS. The dispensability of zinc in bacterial GluRS was already known since a large number of extant (therefore functional) bacterial GluRSs do not contain zinc or a ZB-motif (Table 2). In this context, our conclusion may not seem new, but in the context of *Ec*-GluRS, which natively contains a ZB-motif and which was earlier shown to be zinc-dependent for proper functioning, the conclusion shows that zinc is dispensable even for bacterial GluRSs that are known to contain zinc. This is in addition to zinc being dispensable for many extant bacteria that do contain a ZB-motif.

This not to say that Zn^2+^ doesn't play an important role in GluRSs that contain a zinc-binding motif (like *Ec*-GluRS). Our results show that a zinc-bound *pZBD* may be replaced by a structurally compatible *pZBD* that does not contain a zinc-binding motif without impairing the tRNA aminoacylation efficiency of the parent enzyme. In case of *Ec(Bt)*-GluRS, compatibility between *pZBD*s of *E. coli* (γ-proteobacterium) and *B. thailandensis* (β-proteobacterium) is not unexpected because of their close evolutionary origin (phylum: proteobacteria) and therefore their *pZBD*s are replaceable. On the other hand, the *pZBD*s of *T. elongatus*, a non-proteobacterium (cyanobacterium) and *E. coli* Glu-Q-RS, a paralogue of GluRS, cannot substitute for the *pZBD* of *Ec*-GluRS.

### Alternate structural and functional strategies adopted by GluRS without zinc-bound *pZBD*

**Figure 7:**
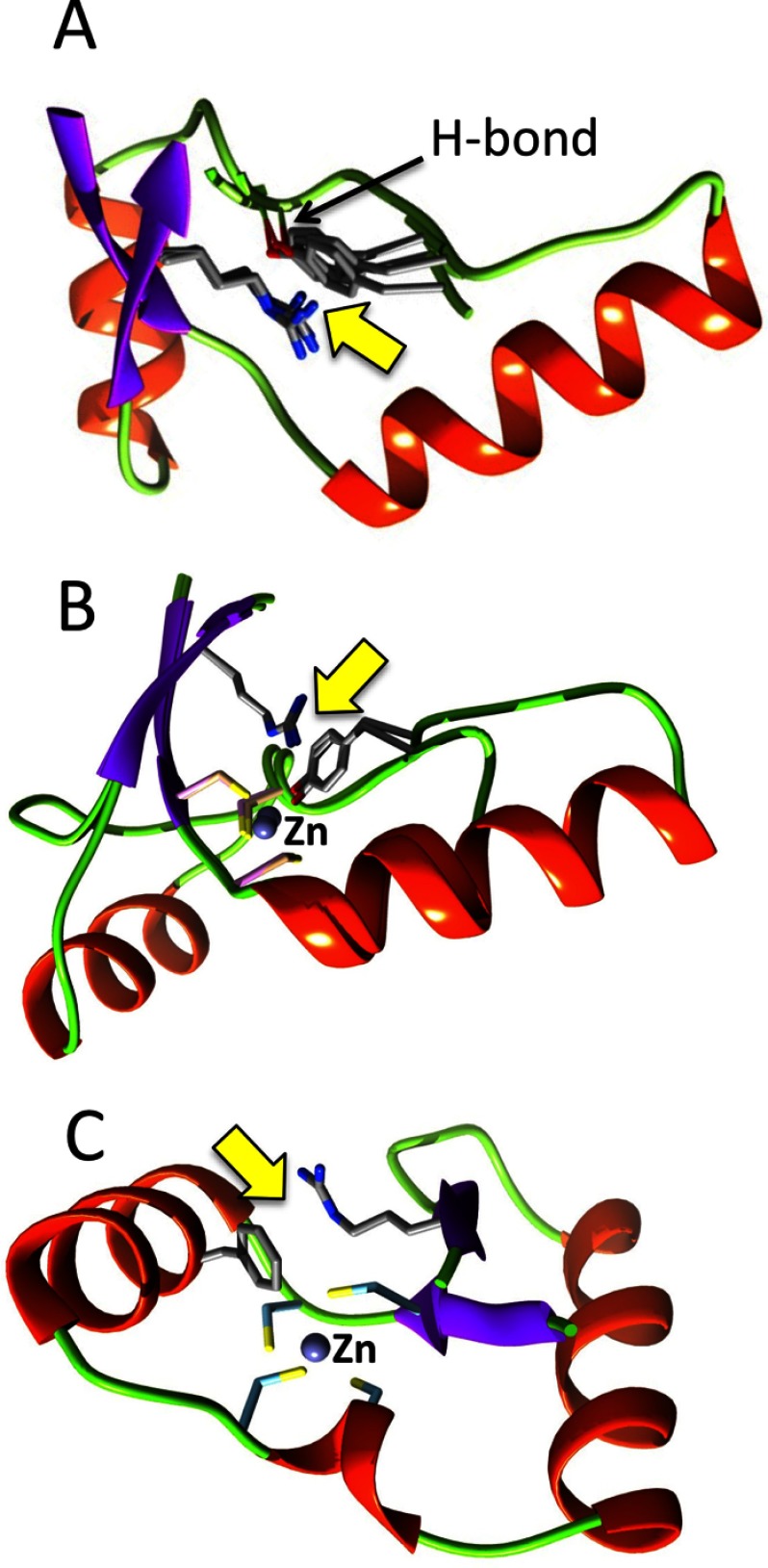
Conserved cation–π interaction in *pZBD*s of bacterial GluRS (**A**) Superimposition of zinc-devoid *pZBD*s from *Mt*-GluRS (2ja2), *Tt*-GluRS (2cuz), *Bt*-GluRS (4g6z) and *Te*-GluRS (2cfo). (**B**) Superimposition of zinc-containing *pZBD*s from *Bb*-GluRS (4gri) and *Ec*-Glu-Q-RS (4a91). (**C**) *pZBD* of *M. thermautotrophicus* GluRS (3aii). Cation–π interactions in each panel [arginine–tyrosine in panels (**A** and **B)** and arginine–phenylalanine in panel (**C**) are highlighted by yellow arrows]. The annotated H-bond in panel (**A**) refers to a conserved H-bond between the phenolic oxygen atom of tyrosine and the (i + 4)-th backbone nitrogen atom.

Structural superposition of GluRS *pZBD*s of a number of bacterial GluRSs (Figure 4) showed excellent overlap of the core fold even though several of the structures lacked a bound Zn^2+^ ion. This indicates that zinc-devoid *pZBD* must use an alternate strategy that provides the intra-chain locking induced by a bound Zn^2+^ ion. An analysis of four zinc-devoid *pZBD*s, from *Bt*-GluRS, *Tt*-GluRS, *Mt*-GluRS and *Te*-GluRS, showed a number of long-range H-bonds (more than four residue separation in the sequence; Supplementary Figure S6), mediated by side chains. In addition, a number of hydrophobic stacking, π–π stacking and cation–π interactions were also observed. We also identified a conserved cation–π interaction in all four structures, between two semi-conserved residues (Supplementary Figure S2), an arginine present in the second β-strand E4 and a tyrosine in the loop joining the helices H4 and H5 that co-ordinates zinc in zinc-bound *pZBD*s (Figure 7A). Interestingly, the tyrosine–arginine cation–π interaction is also present in *Bb*-GluRS and *Ec*-Glu-Q-RS, both containing a bound Zn^2+^ ion in their *pZBD*s. In both, the phenolic oxygen atom of tyrosine participates in zinc-binding whereas the ring π-cloud is found to be stacked with the guanidium moiety of arginine (Figure 7B). The *pZBD* of archaeal GluRS from *M. thermautotrophicus* is also zinc-bound but all four co-ordinating side chains are cysteine residues, with the arginine-interacting conserved tyrosine in bacterial GluRS replaced by one of the cysteines. Despite the lack of a tyrosine residue, the conserved arginine in the second β-strand E4 still participates in an alternate cation–π interaction where phenol, present in the first helix H4, is the alternate π-partner (Figure 7C).

In addition to playing a structural role, the conserved cation–π interaction in *pZBD*s of bacterial GluRS, with or without a bound Zn^2+^ ion, may also be functionally important. In the only tRNA^Glu^-bound structure of *Tt*-GluRS (Figure 1), the arginine (Arg^116^ in *Tt*-GluRS) side chain forms a H-bond with the backbone phosphate of tRNA^Glu^ (Ala^73^). The arginine side chain, locked via the cation–π interaction, is conformationally predisposed for a fruitful tRNA^Glu^ interaction. In *pZBD*s with a bound Zn^2+^, where the phenolic oxygen atom of tyrosine is one of the co-ordinating ligands, the interaction becomes multi-layered. The tyrosine ring, immobilized due to co-ordination with Zn^2+^ ion, in turn, restricts the mobility of the tRNA^Glu^-interacting arginine side chain due to cation–π interaction. Interestingly, in all four Zn^2+^-devoid *pZBD*s, the phenolic oxygen atom of the tyrosine participates in a conserved H-bond interaction with the (i+4)-th backbone nitrogen atom (Tyr^123^–Trp^127^ in PDB ID: 4g6z; Tyr^121^–His^125^ in PDB ID: 2cfo; Tyr^122^–Ala^126^ in PDB ID: 2cuz; Tyr^132^–Asp^136^ in PDB ID: 2ja2). In place of Zn^2+^-ligation, this can be viewed as an alternate strategy for immobilizing the tyrosine side chain. This explains why Zn^2+^ is dispensable in bacterial GluRS [36,37]. The loss of Zn^2+^ ion within the same protein family is rarely observed and when Zn^2+^ is lost, the associated surrounding secondary structure/loops are also often lost [36]. For bacterial GluRS, however the loss of Zn^+2^ does not lead to an overall structural or functional change. It is interesting to note that a zinc-co-ordinating sphere, formed by two cysteines and two histidines, is also absent from many bacterial Ros DNA-binding domains despite the maintenance of a functional fold [37].

### Is facilitation of indirect glutaminylation the core function of *pZBD* in bacterial GluRS?

The *pZBD* of GluRS is functionally coupled with the glutamylation of tRNA^Glu^ [9,10,12]. Therefore, the deletion of the *pZBD* in seven extant bacterial GluRSs (Figure 2E), all wild-type and therefore functional, is surprising. It is possible that the tRNA^Glu^ in these bacteria, especially nucleotides in the acceptor stem, are different to compensate for the deleted *pZBD*s. We compared the tRNA^Glu^ sequences in bacteria for which the corresponding GluRSs (both *pZBD*-containing and *pZB*-deleted) formed a monophyletic cluster. A projection of the seven *pZBD*-deleted GluRS on to a phylogenetic tree of bacterial GluRS (Figure 3) showed that three *pZBD*-deleted GluRSs of tenericutes, *Acholeplasma laidlawii*, *Phytoplasma mali* and *Onion yellows phytoplasma OY-M* are monophyletic with three *pZBD*-containing GluRSs from tenericutes and four *pZBD*-containing GluRSs from firmicutes. Similarly, two *pZBD*-deleted GluRSs from verrucomicrobia and one from planctomycetes, *Methylacidiphilum infernorum*, *Akkermansia muciniphila* and *Phycisphaera mikurensis*, are monophyletic with *pZBD*-containing GluRSs from planctomycetes. The remaining *pZBD*-deleted GluRS (GluRS1) from α-proteobacteria, *Neorickettsia sennetsu*, appears in an isolated clade. Except for the nucleotide identity variation at 11–24 base-pair at the D-helix (AU compared with UA) in selected cases, no systematic differences were observed between tRNA^Glu^ of bacteria with or without *pZBD* in the monophyletic GluRS clusters (Supplementary Figure S7). The D-helix is distant from the *pZBD*-contacting tRNA acceptor stem. Therefore, it can be concluded that tRNA^Glu^ of *pZBD*-containing and *pZBD*-deleted GluRS are not significantly different.

The curious case of these seven *pZBD*-deleted bacterial GluRS becomes more interesting since no such deletion was observed in archaeal and eukaryal GluRSs (Supplementary Figures S3 and S4). Therefore, one may assume that some bacteria-specific evolutionary stress might be responsible for the deletion of this primordial domain. In addition to tRNA^Glu^, bacterial GluRS is evolutionarily and functionally connected with tRNA^Gln^ and the heterotrimeric GatCAB. GluRS glutamylates tRNA^Gln^ in bacteria that lack the tRNA^Gln^-specific GlnRS, yielding the misacylated product GlutRNA^Gln^. The misacylated tRNA^Gln^ is further processed to Gln-tRNA^Gln^ by GatCAB through a ternary transamidosome complex [38–40]. This ancestral route for the production of Gln-tRNA^Gln^ can become redundant when the bacterial genome contains functional copies of tRNA^Gln^-specific GlnRS. In addition, in genomes with two copies of GluRS (GluRS1 and GluRS2), only GluRS2 may participate in the indirect production of Gln-tRNA^Gln^, making GluRS1 strictly tRNA^Glu^-specific [14,31,41,42].

The genomes of all but one *pZBD*-deleted GluRS containing bacteria (*M. infernorum*, the exception, is discussed later) contain either GlnRS or GluRS2 indicating that the indirect glutaminylation pathway, via tRNA^Gln^–GluRS–GatCAB or tRNA^Gln^–GluRS1–GatCAB interaction, may be redundant in them. Of course, one could argue that the mere appearance of GlnRS or GluRS2 in the genome may not necessarily mean that these are functional and that GluRS (or GluRS1) in these bacteria may still, along with GatCAB, produce Gln-tRNA^Gln^ by the indirect route. However, independent genomic signatures clearly indicate that either the tRNA^Gln^–GatCAB or the GluRS–GatCAB or both the interactions are disrupted in these six bacteria. Three genomes (*A. laidlawii*, *P. mali* and *Onion yellows phytoplasma*) lack the gatCAB gene whereas tRNA^Gln^ in five cases (except *N. sennetsu*) lack the characteristic U1-A72 signature (Supplementary Figure S8) required for productive GatCAB recognition [43]. In summary, the indirect glutaminylation pathway seems to be redundant in bacteria that contain *pZBD*-deleted GluRS. The connection between the two becomes evident from the previously solved structure of transamidosome in *T. maritima* [40] where GluRS directly contacts GatCAB through the *pZBD*.

Correlations mentioned above are consistent with the following evolutionary scenario. The *pZBD* of GluRS, present at the N-terminal catalytic domain, is of primordial origin. It appeared before the bacteria-eukarya branching when all GluRS were non-discriminatory, charging both tRNA^Glu^ and tRNA^Gln^ (the *pZBD* is present in extant GluRS from eukarya, bacteria and archaea). There was an evolutionary pressure against its deletion until the genome acquired an alternative route of Gln-tRNA^Gln^ synthesis (acquisition of GlnRS or GluRS2). However, even in the presence of an alternative route of Gln-tRNA^Gln^ synthesis, the *pZBD* may have been retained, since over time, it also played a role in optimizing GluRS–tRNA^Glu^ interaction. Only in rare cases, where deletion of *pZBD* did not significantly impair GluRS–tRNA^Glu^ interaction, it got deleted. This explains why *pZBD* plays an important role in GluRS–tRNA^Glu^ interaction, yet, it is deleted in some extant bacterial GluRS. We hypothesize that the core function of *pZBD* in bacterial GluRS is gatB interaction and not tRNA^Glu^ interaction.

The only bacterium that does not fit this model is *M. infernorum*. Even without GlnRS or GluRS2, it contains a *pZBD*-deleted GluRS (its tRNA^Gln^ A^1^-U^72^ signature also indicates the presence of an active glutaminylation route via GatCAB interaction). *M. infernorum* is an autotroph with an unusually small genome of 2.3 mbp [44] which must be a later adaptation to its unique ecological niche. It is likely that GlnRS was lost during genome streamlining and an alternate mechanism of GluRS–gatB interaction, skirting the use of *pZBD*, evolved. For example, GluRS from *M. infernorum* and *Methylacidiphilum fumariolicum SolV* [45], another thermoacidophilic methanotroph of the phylum verrucomicrobia, also without GlnRS and with a *pZBD*-deleted GluRS, contain conserved cysteine and tyrosine residues in the neighbourhood of the canonical *pZBD* (Supplementary Figure S9). These unique signatures may give rise to alternate structural features that compensate for the absence of *pZBD*. This needs to be further explored by structural and functional studies.

## Online data

Supplementary data
