# Supplementary material for: Dispensability of zinc and the putative zinc-binding domain in bacterial glutamyl-tRNA synthetase
Source: Biosci Rep. 2015 Mar 31;35(2):e00184. doi: 10.1042/BSR20150005 (PMC4381286; doi:10.1042/BSR20150005)
Supplement: Supplementary data [file bsr035e184ntsadd.pdf]

Supporting Information

Dispensability of zinc and the putative zinc-binding domain in bacterial glutamyl-tRNA synthetase

Nipa Chongdar[1,¶], Saumya Dasgupta[1,¶], Ajit Bikram Datta[2], Gautam Basu[1*]

[1]Department of Biophysics and [2]Department of Biochemistry,

Bose Institute, P-1/12 CIT Scheme VIIM, Kolkata 700054, India

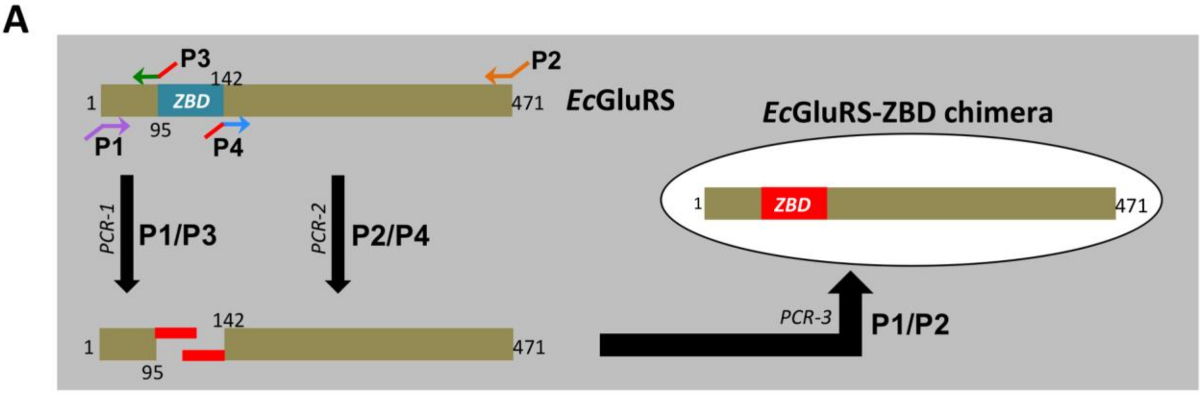

B

**P1**	5′CAGCAGACGGGAGGAGGAATGAAAATCAAAACTCGCTTCGCG 3′
**P2**	5′TCGAGGCCTGGAGCCTTACTGCTGATTTTCGCGTTCAG 3′

**P3**	5′GTTCCGGCAGCACTTTGCCCGGTTCCGGGCGCCAGGTACCGTCATAACGCGGCTTC 3′	***Ec(Bt)*-GluRS**
	5′ATCATAGCGCGGCGCCTGGCCTTTCGCTTTCTGTTCCGCGCGCAGCGCTTCCAGTTCTTCCG GGGTGCAATAGCAATAATAAGCAGTGCCCTCTTCCAG 3′	***Ec(Te)*-GluRS**
	5′ATGGCCATCATAAATGCCGCCAATGCTCTGAATGCGCGCGCGGGTGCAGGTGCAATAATAA GCAGTGCCCTCTTCCAG 3′	***Ec(EQRS)-GluRS***
	5′GCCGATGCTCTGGATGCGCTCTTTAGAGCAATAGCA 3′	***Ec(ΔH4)-GluRS***
**P4**	5′CAAAGTGCTGCCGGAACCGCCGGCGGGCGTGGCGCCGGTTGTACGTTTTGCTAACCCGCA 3'	***Ec(Bt)*-GluRS**
	5′GCGCCGCGCTATGATAACCGCCATCGCCATCTGACCCCGGAAGAACAGGCGGCGTTTGAAG CGGCGGGCCGCACCCCGGTGGTACGTTTTGCTAACCCGCAG 3′	***Ec(Te)*-GluRS**
	5′ATTTATGATGGCCATTGCCGCGTGCTGCATCATGGCCCGGATAACGCGGCGGTACGTTTT GCTAACCCGCAG 3′	***Ec(EQRS)-GluRS***
	5′AGCATCGGCGGTATCTATGACGGTCGCTGCCGC 3′	***Ec(ΔH4)-GluRS***

Supplementary Figure S1. Construction of *pZBD*-chimeras.

(**A**) Schematic of PCR-based cloning. (**B**) List of primers used.

Group-I: CxCx$_{20\text{-}21}$Yx$_3$C

```
                                                                       *                                               *
ECO_ga   AYKCYCSKERLEAL---REEQMAKGEKPR------YDGRCRH-------SHEH-----HADDE----PCVVRF
MSU_ga   AYRCYCSKERLEEL---RHQQEANKEKPR------YDRHCLH-------DHE------HSPYE----PHVVRF
GLO_de   AYRCWCRPEELEAK---REAAMAEGRKPK------YDGTCRH-------------RQDQPLDQ----PHVIRF
AFE2_ga  AYACYCSADDLAAE---RAVQRSAGKAPR------YGGRCRH-------LDAAARAEREAAGL----LPTLRF
AEH2_ga  AYPCFCTERELELS---RKAQRAAGKPPR------YAGTCAH-------LTAEERERRRAEGR----RPTLRF
TGR2_ga  AYPCFCTERELELS---RKAQRAAGKPPR------YAGTCAH-------LTAEERERRRAEGR----RPTLRF
MCA2_ga  AYPCFCTPLELEVS---RKVQLGSGRPPR------YSGRCAH-------LPADEVRRRHEEGL----AATLRF
TGR1_ga  AYPCFCSEASLKLS---RKAQLAAGQPPR------YPGTCSH-------LSPDEVQARLAQGL----KPTLRF
MCA1_ga  AYRCYCTKEELDEL---RAGQMERKEKPR------YDGRCRH-------RSE------PRPGV----LPVIRF
TAM_ht   VYECYCTQEELEQM---RKEQLERGEPPR------YTGKCRH-------LTEAEKEKLRAEGR----KPVLRF
LMF_fi   AYKCYCTEEELDAE---REKQKANGEMPR------YSGKCRH-------LTKEQQAEKEAQGF----KPSIRF
MHD_dt   AYRCFCTPERLEAL---REEQKKRGLPLG------YDGHCRH-------IPPEEAEARAKAGE----PHVVRL
AAE_ht   AYPCFCTPEELEKE---REEARKKGIPYR------YSGKCRH-------LTPEEVEKFKKEGK----PFAIRF
ATM_ns   AYYCFCTPERLEKV---RQEQQARKEQPH------YDGTCRH-------IDPDEAARRVARGE----RHVIRF
PCU_ch   AYKCFCTAAELDEM---RELSAKQGGRQG------YDRRCRH-------LTPDEIIEREQAEL----SYVIRL
AVN_ga   AFPCFCSAERLDEV---RARQMANKETPR------YDGHCVH-------IAAEEAQRRIAAGE----SHVVRM
PAE_ga   AFTCFCTPERLDAV---RAEQMARKETPR------YDGHCMH-------LPKDEVQRRLAAGE----SHVTRM
ATU_al   AFRCFCTPERLEQM---REAQRAAGKPPK------YDGLCLH-------LKAEEVTTRVAAGE----ANVVRM
DPR_de   AYKCFCTKEELEQK---REAAMAAKQRDV-----GYDGTCRH-------LTAEEIQAKEAQDI----PATIRF
APL_ga   AYRCYCSKERLENL---RHEQEANKEKPR------YDRHCLA-------HHD------QPTDA----PHVVRF
FTT_ga   AYYCSCSKERLEEL---REYQQANNLKTG------YDGKCRD-------ANYI-----PQQGE----SYVVRF
HDU_ga   AYRCYCSKDRLEQL---RNEQEASKEKPR------YDRHCLN-------HQD------RSINE----PHVVRF
PMU_ga   AYRCYCSKERLEDL---RNTQEQNKQKPR------YDRHCLG-------DHK------HSPEQ----PHVVRF
AHA_ga   AYKCYCSKERLEAL---REGQMANGEKPR------YDGKCRD-------HAHD-----HPADA----PHVIRF
ILO_ga   AYKCYCSTERLEKM---REEQMAAGEKPR------YDGHCRD--------------NPNVGGD----KYVIRF
PLU_ga   AYRCYCSKEHLEAL---RETQMANGEKPR------YDGRCRD-------NPCQ-----HDPAQ----PHVVRF
PCA_de   AYRCYCTAEELEKK---REAAMQDGRKPK------YDGTCRQ-------------LQEVCDDK----PYVIRF
DAK_de   AYKCFCSKEELEAR---REAAVKAKADYR------YNGACRDL-----SPEEAAA--REAAGT----PYVIRF
SAT_de   AYYCTCTPDELEEK---RKRALAEGRKPK------YDGTCRE--------------KKLPPSP----GTVVRF
DAO_de   AYYCNCPKEELEQR---RQAALARGEKPR------YDGHCRD--------------LGLKAGS----DTAVRF
DBR_de   AYWCHCSPETLQAK---REAAMASGAKPM------YDGCCRG--------------KGLGPAP----GAVVRF
SFU_de   AYYCDCAPDDLERR---RKSAMAEGRKPK------YDGRCRD--------------RGLGPGP----DRVLRF
DOL_de   AYYCTCSPERLKER---RERALAEGRNPT------YDGTCRE--------------KALPPSD----DAVVRF
DAT_de   AYYCSCSPEEVDAM---REEAKANGKRPM------YNQRCRN--------------LDLGPGE----NRVVRL
DAL_de   AYYCTCTPEELDEK---RKAAMARGDKPK------YDGTCRE--------------KGLEKCE----GAVVRL
HMR_de   AYRCYCSKERLERL---KEEQLKRGENPH------YDGHCRN-------LNE------YPEDK----PYVIRV
DVU_de   AYWCECPPDEVEKM---REEARAKGLKPR------YNGRCRS--------------RDLGPGD----GRVVRL
LIP_de   AYWCHCSIEQIEKM---REEARQKGEKPR------YNGCCRE--------------KKLSANP----SGVVRL
DRT_de   AYYCQCTPDEVEAM---REQARARGDKPK------YDGRCRE--------------KNLGPGP----DRVVRF
ABU2_ep  AFACFCSDEKLEEL---REESIKKGIPFR------YDGFCENLSDEAV--------LNVNA------PFTVRL
CJR2_ep  AFACFCTEEELEAK---KELAKKQGKAYR------YDGTCEKLADIDV--------LECEK------PFVIRL
HHA2_ga  AYPCFCSERELEMA---RRAQRSAGQPPR------YPGTCAR-------LTAEEVARKRAEGM----QPALRF
NOC2_ga  AYPCFCSQEELERV---RKRQLAAGQAPR------YPGTCAR-------LSPEEVEGKLAAGF----KSALRF
CBU2_ga  AYPCFCSEEQLRLS---RKIQRSAGKPPR------YAGTCRS-------LSAAEIEKKKAEGL----QPALRF
AFE1_ga  AYHCYCSREEVEAM---REDQRQRGEKPR------YDGRCRE-------RTT------VPEGV----APVIRF
HHA1_ga  AYYCYCTKDRLERL---RTEQQARKEKPR------YDGRCRD-------LDG------PPSEEV-ADEPVIRF
AEH1_ga  AYRCYCSKERLDAL---RQEQMANKQKPR------YDGRCRD-------LTE------PPEGA---GEPVIRF
NOC1_ga  AYLCYCTREELASM---RAEQMARKEKPR------YDGRCRE-------RHS------PREGV----SPVVRF
CBU1_ga  AYRCYCSKERLIEL---RNTQLKNKQKPR------YDGFCRD-------KAP------RQSNE----PFVIRF
ECH1_al  AYYCYCSEEEINKE---KEEFSKKGLY--------YKHNCIWKN-----KNFTI------DNL----TRVIRL
MPN_te   AYRCFCTKEKLEHE---RQLALEHHQTPK------YLGTCRN-------LHSKHIQTNLDNQV----PFTIRL
UUE_te   AYFCFCSKEQLDAD---RELAEKSHQTPK------YKRHCLN-------LDKKTIESNLLQNK----EYTIRL
SCO_ac   AYRCYCSQEELDTR---REAARAAGKPSG------YDGHCRE-------LTDAQVEEYTSQGR----EPIVRF
BPJ_sp   AYYCFCTSEELEKK---SNMQKTLNQPII------YDGKCKD-------IPLEEAKRRVANGE----PAKIRF
DTE_ht   VYECFCTPEELDAM---RKEQLERGEPPR------YTGKCRN-------LTEKEKERFKAEGR----KPVLRF
EFA_fi   AYKCYCTPEELEAE---REAQQARGEMPH------YAGTCAN-------LTPSEQAAKEAAGL----EPVIRF
DDF_ht   AYKCFCSKEELEEK---KERALKEGKPPR------YNGKCRN-------LNENQIEELEKKGV----KPSIRF
MFL_te   AYRCFCTTEELEKD---YEDQVAKGIVAT-----KYSGKCSR-------LSESEIESNLKSNK----DFSIRF
IAL_gs   AYYCFCSAERLEAL---REEQQKQKLPQA-----KYDKHCLY-------LSKSEIEANLEKGI----PKVIRL
SSM_sp   AYYCFCSSERLETL---RQEQQKSKASRV-----GYDGHCRS-------IPLEEAKQRIAAGE----HPVVRF
LBA_fu   AYYCFCTAERLQKL---RERQAAMKQAPG------YDGHCRN-------LSKEEVEAKLAAGE----PYVIRL
HOH_de   AYRCFCTSERLAEM---REERRAQKSNIT-----GYDGRCRG-------IEAAESERRAAAGE----PFVVRL
FNU_fu   AYYCFCDHERLENL---RERQKAMGLPPG------YDGHCRS-------LSKEEIEEKLKAGV----PYVIRL
STR_fu   AYYCFCTPERLSKL---RERQIAMKQAPG------YDRHCRN-------LTDEEVKQKLAEGV----SYVIRL
DTH_ht   AYYCYCTPEELEER---RKEALAQKKPPR------YDRKCLY-------LSDEERARYEREGR----KPAIRL
TAL_ht   AYPCFCSPEELEKE---RQEAYQKGIPYR------YSGKCRY-------LTKEEAEELKKKGK----PFAIRF
AOE_fi   AYYCFCSKERLDEV---REKQKVEGLNAG------YDGHCRN-------LSKEEIEEKLNAGQ----PYVIRL
FMA_fi   AYYCFCSKERLDQV---KAQQKADGLMPK------YDGLCRG-------ISIEDAKKRIANGE----EYVIRL
TID_ht   AYYCECSPEVLEEK---RKKALAEGRKPK------YDGTCRE--------------KGLGPGP----GRVLRF
NDE_ht   AYWCVCTSEELEAR---RKEAETKGGSPK------YDGRCRN-------------LGLTKPTG----EAALRF
DET_ns   AYYCHCSSERLDKM---REEQIARKEPPG------YDRCCRD--------------LCLGQKE----GAVIRF
DLY_ns   AYRCTCSPGRLEAM---RAEQTAAKKPPG------YDRCCRN------------RTEPVAQGV----ASVVRF
PUV_ch   AYKCFCTAEELSEM---RELLAKSGGRQG------YDRRCRN-------LSSEEITEKEAAGL----PYVVRL
```

```
WCH_ch  AYKCFCTAEELAEM---RQVLAKKGGRQG------YDRRCRN-------LSPEEIKEKEAAGK----PSVIRL
SMD_al  GFRCFCTPERLEQM---REAQRAAGKPPK------YDGLCLS-------LSAEEVTSRVAAGE----PHVVRM
RET_al  AFRCFCTPARLEQM---RETQRAAGKPPK------YDGLCLN-------LTAEEVTARMAAGE----TTVIRM
```

(continued in next page)

```
LAS_al  AFRCFCSTERLEEM---RRAQRKRNIPSR------YDGHCLK-------LSSQEIERLSQEQK----KHVVRL
SFD_al  GFRCFCTPERLEQM---REAQRAAGKPPK------YDGLCLS-------LSAEEVTSRIAAGE----PHVVRM
PUB_al  AYKCYCSSAEIEEQ---KKRARQKKLPYV------YNRKCRD-------LQETN----APTII----KPVIRF
MXA_de  AYRCYCTKEDLDAQ---RQVAEKAGGAFK------YPGTCRE-------RTEPPA---GRNAA----DAVIRF
SUR_de  AYRCYCTQEEIRER---RAQAEKEGRAYK------YEGTCRE-------RKDVPEGR----------PSVVRF
ADE_de  AYACYCTREELDAQ---RKQAEAEKRQFR------YPGTCRD-------------EPYDPSR----PHVVRF
AFW_de  AYACYCTKDVLDAQ---RKQAEAEKRQFR------YPGTCRE-------------LPYDPSR----PHVIRF
DPS_de  AYKCFCSKESLDEK---REALAAKKSLG-------YDGTCRN-------LTAEQIAEKEAAGA----PYVLRF
ABA2_ad AYYCFCSQADLDAY---REQALKDHRPPI------YPGTCRS-------VDPAEAKRRRDSGE----AGAIRL
ACA2_ad AYRCFCTQEDLEAE---RARAVAEHRPQV------YSGRCRA-------LSAETSAQRAAAGE----PFAVRL
ACA1_ad AYHCFCTKEELEQR---RAEATAAGRPPM------YDRRCRR-------IAPSDAAARKAAGE----PAALRF
ABA1_ad AYYCFCSKEGLEQR---RKAATAAGRAPQ------YDESCRK-------IGREDAAARKQGGA----PCAVRF
```

Group-II: $CxCx_{20}Yx_3H$

```
                                           *                                 *
ECO_ga  AYKCYCSKERLEAL---REEQMAKGEKPR------YDGRCRH-------SHEH-----HADDE----PCVVRF
TEL_cy  AYYCYCTPEELEAL---RAEQKAKGQAPR------YDNRHRH-------LTPEEQAAFEAAGR----TPVIRF
CYT_cy  AYRCYCTPEELEQM---REAQKAQNQAPR------YDNRHRH-------LTEEQRQGLEAQGR----KPVIRF
SYF_cy  AYRCYCTEAELEAL---RESQRARNEAPR------YDNRHRD-------LTPEQEAAFQAEGR----EAVIRF
BSS_fi  AYKCYCTEEELEKE---REEQIARGEMPR------YSGKHRD-------LTQEEQEKFIAEGR----KPSIRF
LIE_sp  AYRCFCTQEELEAK---KKQSEAMGVPYV------YDGLHAN-------MSDEEVQEKLKQGI----PYSVRF
```

Group-III: *No ZB-motif (long pZBD)*

```
                                           *                                 *
ECO_ga  AYKCYCSKERLEAL---REEQMAKGEKPR------YDGRCRH-------SHEH-----HADDE----PCVVRF
NMU_be  AYYCYCSREELDAM---REQQRAAGLKPR------YDGRWRD-------SREE-----PPAGV----KPVVRL
RRU1_al AYRCYCTPDELTAM---REEQKAKGLPPR------YNGLWRD-------RDPSE----APAGV----APVIRL
MMR1_al AFKCYCTAEETQLL---RDEAFAAGRALR------SP--WRD-------RDASE----ARADT----PFTIRF
MCT_ga  AFRCFCTPEELDAM---REAQMAAGLPVK------YDGRYAN-------LSREESDALVAQGK----PFVIRM
MAR_cy  AYRCYCTPEELEKM---REEQKARNLAPR------YDNRHRY-------LTPEQQAQFEQAGR----KAVIRF
BBR_be  AYHCYCSPEEVDAM---REAARAKGLKPR------YDGTWRP-------EPGKTL-PPVPADR----KPVIRF
TBD_be  AYHCYCSTEELDEM---REAQRARGEKPR------YDGRWRP-------EPGKTL-PVPPSGV----QPVVRF
MFA_be  AYHCYCSREELDAL---REQQMREGKKPR------YDGRWRP-------ESGKVL-PAIPADI----PPVVRF
NMC_be  AYYCYCSKEELEAM---REKAEKEGTAT-------YDRRWRP-------EAGKTL-PEIPAGV----QPVVRF
WSU2_ep AFYCYCSKEFLDQK---REEALAQKLPFR------YHDAWAE-------------IEKDSTQ----KPVIRL
CTH_fi  AYPCFCSEEELEEI---RKQQLAENVNTG------YYGKWAVH----RNLTLEEVQKHLENNE----SFVIRF
APM_al  AYKCYCSNEEIEEQ---KERARQKKIPYI------YNRKWRE-------LDEKD----APKDI----KPVIRF
AEX_al  AYPCFCSPEELDEL---RTRQQLRSVAPG------YYGEWAIW----RHKSLEEALEALKAGK----PYVIRF
MTU_ac  AYHAFCSTPEEVEAR---HVAAGR-NPKLG------YDNFDRH-------LTDAQRAAYLAEGR----QPVVRL
OCA_al  AYRCYATPEELTKM---REAARAEGRAVR------YDGRWRD-------RDPSE----APADV----KPVIRL
BJA_al  AYRCYATAEELTAM---REKARAEGRTRL------YDGMWRD-------RDPAT----APSDV----KPTIRL
NHA_al  AYRCYATPEELAEM---REKARAEGRAKL------YDGRWRD-------RDPSE----APPDL----KPTIRL
RPD_al  AYRCYATAEELTAM---RDKARAEGRSKL------YDGSWRD-------RDPSD----APADL----NPTIRL
RFR_be  VYPCYMSVAELDAL---RDAQMAAKEKPR------YDGTWRP-------APGKTL-PPIPEGV----KPVLRF
RME_be  AYHCYMSTEELDAL---REAQRAAGEKPR------YNGFWRP-------EPGKVL-PEPPAGV----QPVVRF
DAR_be  AYYCYTTREELDAL---RAEQEAKKEKPR------YDGRWRP-------EAGKAL-PVPPTDV----PPVIRF
RSO_be  AYPCYMSTEELDAL---REAQRVRGEKPR------YDGTWRP-------EPGKVL-PTPPAGV----QPVIRF
LHK_be  AYYCYMTREELDAL---RAEQEARGEKPR------YDRRWRP-------EPGKEL-PAPPAGV----TPVVRF
PZU_al  AYRDYMTPEELEAE---REVARAEGRVVR------SP--WRD-------ASPN-----DAPDR----PFVVRL
CCR_al  AYRCWMSIEELEVA---REKARAEGRAIR------SP--WRD-------APEG-----DLSA-----PHVIRF
HPY2_ep AFYCYASTEFLERE---KEKAKNEKRPFR------YLDEWAA-------------LEKDKHH----APVVRL
NIS1_ep AYYCYMSKEELDRL---REEQMKRGERPR------YDRRYRD-------FTG-----TPPQGV----QPVVRI
CJR1_ep AYYCYMSKEELEEL---RAKQEAAKERPR------YDGRYRE-------FTG-----TPPQGI----EPVVRI
HPY1_ep AYYCYMSKDELDAL---REEQKARKETPR------YDNRYRD-------FKG-----TPPKGI----EPVVRI
WSU1_ep AYYCYMSKEELDAL---REEQRSRGETPR------YDNRYRD-------FTG-----TPPSGV----APVVRI
ABU1_ep AYKCYMSKEELDAL---RAAQEAAKQTPR------YDGTWRP-------EPG-KELPPVPAGV----EPVIRI
TDN1_ep AYRCYMSREELDAL---RETQMANKERTK------YDGKYRD-------FDG-----TPPDGV----DSVIRI
SKU1_ep AYKCYMSKEELEAL---REEQTARKERPK------YDNRYRD-------FSG-----IAPEGR----EAVIRI
SDL1_ep AYKCYMSKEELDAL---REAQMARKERPR------YDGRYRN-------FTG-----TPPAGV----EPVIRI
SUN1_ep AYKCYMTKEELNAL---REEQMAKKERTR------YDGRYRD-------FTG-----TPPEGV----KPVIRI
NSA1_ep AYKCYMSKEELDAL---RAGQMARGERPR------YDGRYRD-------FTG-----TPPEGV----APVIRI
OAN2_al LYACYETADELERR---RKLRLARRLPPV------YGREGLK-------LTDDEKAAFEAEGR----KPHWRF
BMC2_al LYACYETADELERR---RKFRLARRLPPV------YGREALK-------LTDAEKAALEAEGR----KPHWRF
MMR2_al LYPCYETGEDLDRK---RRLQMANGRPPV------YDRAALD-------LTDAEKAGFEAEGR----KPHWRF
MLO2_al LYACYETPEELDLR---RKVRRTRGLPPV------YGREALA-------LTHEQVAEYQADGR----RPHWRF
RRU2_al LYACYETPEELEYR---RRRQRAQGLPPI------YDRAGLA-------LSAAERAALEAEGR----KPHWRF
RSP2_al FYECFESPTELDLK---RKKLLNMGKPPV------YDRAALK-------LSDEERARLRAE-R----GGYWRF
PDE2_al LYEVFETPTELDLK---RKKQLNMGKPPV------YDRAGLK-------LSAEDKDRLRAEGR----AGYWRF
```

```
NAR2_al IYRAYETAQELDLK---RKILLGRGLPPI------YDRAALK-------LTEADHAAKAAGE----RPHWRF
ECH2_al IYPCYESKEELEFK---RKMKLKLGLPPI------YDRSALN-------LTQAEKDKYSE--R----APYFRF
OTS2_al LYECYETPEMLEIE---RKRQLTSGYPPI------YSRKALE-------LTKAQKVQLQAEGY----KVHYRF
NSE2_al IYPCYETPEELEIE---RRSLLARALPPV------YRRKN----------------RPQHSTR----LPYYRF
RPR2_al LYACYETKEELELK---RKLQLSKGLPPI------YDRASLN-------LTEKQIQKYIEQGR----KPHYRF
OAN1_al AYYCYATPEELAEM---REKAREEGRPPR------YDGRWRD-------RDPSE----APAGV----KPVIRI
BMC1_al AYYCYASPEELEEM---REKARAEGRPPR------YDGRWRD-------RDPSE----APAGV----KPVIRI
PDE1_al AYKCFSTTEEIEAF---REQAKAEGRSTL------FLSPWRD-------ADPAG-----LPDA----PYAIRL
RSP1_al AYKCFSTQEEIEAF---REAARAEGRSTL------FRSPWRD-------ADPTS-----HPDA----PFVIRM
MLO1_al AYYSYETPAELEAM---REAARAKGLPPR------YNGQWRD-------RTPSE----APAGV----KGAIRI
OTS1_al AYYCFTSPEEIDLQ---RQLAITQKQSFI------FRSPWRN-------NIPSS----LSLKNN-NKAYVIRF
NAR1_al AYRCYLTQEELAAR---RELAQAERRPFR------IDSEWRD-------ATPDQ----WPADQ----SYVVRM
RPR1_al AYYCFTRQEEIAKQ---RQQALKDKQHFI------FNSEWRD-------KGPST----YPADI----KPVIRL
BFR_ba  AYIAFDTPEELDAK---RAEIANFQYDAST---RVGMRNSLT-------LPKEEVEALIADGK----QYVVRF
PGI_ba  AYIAFDTPEELEAR---RAEVPNFQYDATT---RGQMRNSLT-------LPAEEVERLVAEGT----QYVVRF
PDI_ba  AYIAFDTPAELEEK---RKEIANFQYDAST---RSQMRNSLT-------LSQEEVQSLIESGH----QYVVRA
COC_ba  AYYAFDTAEALDTA---RKEAEAKGETFI------YNWATRDALQNSLSLSKEEVQQRLERGD----EYVIRF
GFO_ba  AYYAFDTSDELDGH---RKDHEEKGKTFI------YNWHNRQKLKNSLSLSQEEVQEKLDGEE----DYVIRF
FJO_ba  AYYAFDTPEALDAH---RKQHEAEGKTFI------YNHHNREKLDTSLVISAEETAKRIANGE----HYVIRF
RAN_ba  AYIAFDTPEELDAI---RKEFESRGEVFA------YNYRTRGRLRNSISLPKEEVEQLLAENT----PYVVRF
SRU_ba  AYYAFDTEEELEEL---REEHGAYDGSTR-----RQMRNSLT-------LSDEKVEQRIDEGD----DYVIRL
RER_ac  AYESYSTNEEVDAR---HKAAGR-DPKLG------YDNFDRD-------LSDEQRAAYVAEGR----KPVVRL
CGL_ac  VYPAYSTAEEVEER---HKAAGR-DPKLG------YDNFDRD-------LTEEQVAAFEAEGR----KPVWRL
RSA_ac  VYESYSTPDEIEAR---HKAAGR-DPKLG------YDGFDRD-------LSAEQIAAFKAEGR----EAALRL
MLU_ac  IYPSYSSPEETEAR---HRAAGR-DPKLG------YDGHDRD-------LTDEQIAAFEAEGR----APVWRL
BAD_ac  AYESFSTPEEIEAR---NVAAGR-PKAFG------YDGYDRN-------LTEEQKAAFRAEGR----KPALRI
IPA_pl  VYKDFSTEDERAAD---KAAAEARKQPYR------FRRKPID---------PATLAQFEAEGR----PYALRF
PLM_pl  AFKDFDAPEVVQQD---RAEAEAAKRNYV------NIRRSLE-------LTPEKIAELEAAGT----PFVVRL
PSL_pl  AYYDYGRPEELKAE---REAADAAKRPFL------YSRQWMA-------TTPEERAKFEAEGR----SFVVRL
GOB_ac  AYESFSTNEEVEAR---RLAAGQ-DPKLG------YDNADRF-------LTDEQKAALRAEGR----EPVLRL
RBA_pl  AYRDFAKPEELQTL---REEAQKGGEAFV------YDRRWMA-------EDEATAAKFEAEGR----QGVVRL
OOE_fi  AYKSYKTEATLKEE---REAQ-AQAHQAP-----HYVYEYAG-------MTKEQIKDAQAKDEAAGLKSVVRF
CLI_gs  AYYCFSTSEELEEN---RQLQLKQGLQPK------YNRKWLPEEM-GGAMPSSNIRKKMAEGS----PYVVRM
CTE_gs  AYYCFSTPEELEEN---RQLQLKQGLQPK------YNRKWLPEDM-GGNMPESEIKKKLDEGA----PYVVRM
PLT_gs  AYYCFATSEELEEN---RQLQMKQGLQPK------YNRKWLPEDM-GGSMPRSESEKMLASGA----PYVIRM
PAA_gs  AYYCFSTAEELDEN---RKLQMKQGIQPK------YNRKWLPETM-GGNMPQSEIQKKLDEGA----PYVIRM
TTH_dt  AYRAFETPEELEQI---RKE---KG---G------YDGRARN-------IPPEEAEERARRGE----PHVIRL
DRA_dt  AYYAFETSDELAAL---REEAQKAGHVI--------AIPSRD-------LGAAQAQARVDAGE----PAVIRL
MRB_dt  AYRAFDTPEELAAA---REAAQRAGHKEQ-----GYNRRYRD-------YPVEEAERRAAAGE----PHVVRL
TRA_dt  AYRAFETPEELEAI---RLELQAKGLGHG------YDGRARA-------LSKEESDRRAAAGE----PFAVRL
OPR_dt  AYRAFETPEELAAI---REELQRKGLGYG------YDGRARK-------IPREEAEARATAGE----PHVIRL
SMF_fu  AYYSFETPEELEIM---RERQKAMGLPPM------YDRRSRN-------LTKEQVEENLAKGL----PYVIRL
SMG_ba  AYYAFEKKKKKRNY---NRKKLDLN------------------------ISNEKTFKKIINGI----VFVIRL
PMC_cy  AYRCFTSESEISEL---REKQKSSGLPPR------HDNRHRN-------LTSNEIKAFLSQGR----SSVIRF
NPU_CY  AYRCYTTSEELEAL---REAQKARGEAPR------YDNRHRN-------LTPEQRAAYEAEGR----SYVIRF
NAZ_CY  AYRCYTTFEELDAL---RETQKARNEAPR------YDNRHRN-------LTPEQRAAFEAEGR----SSVIRF
RCA_ns  AYLSFTTEEELERM---RAEAQARGVKAF-----RFRGPERD-------WPLDRQREMAATGR----PYTVRL
CAG_ns  AYMSFTTEEELTQM---RAAAEAAGIKAF-----RFRGSERD-------WPLERQRELAASGK----PYTVRL
CTR_ch  AYKCFATPQELQEM---RAVASTLGYRGG------YDRRYRY-------LSPEEVRQREEQGQ----PYTIRL
CPA_ch  AYKCFATPQELAEM---RAVASTLGYRGG------YDRRYRY-------LSPEEVASREAAGQ----PYTIRL
SNG_ch  AYKCFATAAELKEM---REIAAKTGQRSG------YDRRYRN-------LSPQEIEKRESEGQ----SYTIRL
MAQ_ga  AFYCFRTPEELDAI---REERKAQGLNPG------IKG-DLE-------LPEEEVKRRLDAGD----PYVIRM
GPB_ga  AFYCFATPDELDEM---RREQQARGETPR------YDGRGLE-------LSPSEVQRRLGAGE----PHVIRM
ACI_ga  AFYCFATAEELDQM---RAEQQARGETPK------YDGRGLK-------LSQEEVERRLAAGE----PHVIRM
HCH_ga  AYYCFRTPEELDVI---REERKAQGLPP-------GIKGDLE-------LPDDEVKRRLAAGE----PHVIRM
TTU_ga  AFKCYRTTEELETL---RAERKEAGIHSA------LKPSDLR-------LSEEEQHAREAQGM----PYVVRM
BBA_de  AYYCFMTEEDIEKQ---KAAAGSFAHLV---------SPYQD-------WTLDQALERLKTGD----KAVVRF
TAF2_ht AYKVYAYPEEIEKL---REELLSQGKAPH------YTREMLES-----FTTPERIKEYEEKGL----KPAIYF
FNO2_ht AYEVYAYPEEIEQL---REKLLSEGKAPH------YTREMLEP-----YNTPERKKEYEEKGL----KPAVYF
TMA2_ht AYYVYAYPEEIEEM---REKLLSEGKAPH------YSQEMFEK-----FDTPERRREYEEKGL----RPAVFF
PMO2_ht AYEAYISPEEIEEV---KNQLISEGKPPH------YTYDLISK-----YNTKERIEEYNYKGL----KPVIFL
KOL2_ht AYPVYAYPEEIEKI---HDELLAQGKPPH------YSEELFKQ-----FDTPERRKELEERGL----QPAIFF
```

Group-IV: *No ZB-motif (short pZBD)*

```
                                           *                                      *
ECO_ga  AYKCYCSKERLEAL---REEQMAKGEKPR------YDGRCRH-------SHEH-----HADDE----PCVVRF
CAA_ve  TYEKDGAIWFKLEG------------ER-------YTEYDDF-------------------KK----AEVEKV
OTE_ve  TYEKDGAIWFKLLG------------ER-------YEVFDEH-------------------RK----KTVTKV
KOL1_ht AYYAIYHKDDPKTV---IKTTTEEP-------------------------------TDLGEEY----TYTVNF
PMO1_ht AYFSVTKNDEIIFE---GNNLLDK--------------------------------YKKNNDY----SVVVKF
FNO1_ht AYFAIYDENDPKKV---IEKTTKE--------------------------------PKTKNPF----TVV--F
TAF1_ht AYYAVYHGQEEIEQ---SYEFPEK--------------------------------YANKENF----SIVVKF
TMA1_ht AYYVVYDKEDPSKELFTTYEYPHE--------------------------------YKEKGH-----PVTIKF
```

Group-V: *No pZBD*

```
                                    *                                    *
ECO_ga   AYKCYCSKERLEAL---REEQMAKGEKPR------YDGRCRH-------SHEH-----HADDE----PCVVRF
NSE1_al  AYHD---------------------------------------------------------------NGAVRL
MIN_ve   AYIK---------------------------------------------------------------DEAVYF
AMU_ve   VYED---------------------------------------------------------------EGAWRF
POY_te   AYRDFEKDKK---------------------------------------------------------NFAIRF
PML_te   AYKEYKTEDNQ--------------------------------------------------------KYSLRF
ACL_te   AYKDFKEGSE---------------------------------------------------------DFAIRF
PHM_pl   AYERDGA-------------------------------------------------------------VLFRF
```

Supplementary Figure S2. ZB-motif-based classification and sequence alignment of *pZBDs* of bacterial GluRSs (see Table S1 for abbreviations; *E. coli* GluRS shown in each group for reference where the Zn-binding amino acid residue are marked in red). The conserved Tyr and Arg residues that participate in cation-π interaction are highlighted (green).

Group-I: **CxCx$_{11\text{-}28}$Yx$_3$C**

```
ECO GluRS   AYKCYCSK----ERLEA--------LREEQMAKGE--------KPRYDGRCRHS-------HE-------------HHADDE-
PCVVRF
ECO         SYYCTCTR----ARIQS--------IG-----------------GIYDGHCRVL-----------------------HHGPD-
NAAVRI
MCA         VFPCTCPR----RELAG--------SG-----------------PIYSGRCRAR----------------------
YPHPPAGEHALRL
PIN         CYACACSR----KIIRQ--------SG-----------------GLYQGTCRNK-------QL--------------PEINH-
ALRINL
KPN         SYYCTCTR----ARIQS--------IG-----------------GIYDGHCREL-----------------------CNGPQ-
QAAVRI
SPE         SYCCNCTR----SRIQQ--------LG-----------------GLYDGHCRHL-----------------------NLGPQ-
GAAIRL
SGL         SYYCDCTR----SRIQS--------LG-----------------GLYDGHCRDQ-----------------------HLSAA-
GAAIRL
SDY         SYYCTCTR----ARIQS--------IG-----------------GIYDGHCREL-----------------------HHGPG-
NAAVRI
STT         SYYCTCPR----SRIQR--------LG-----------------GIYDGHCRTL-----------------------CHGPE-
NAAVRI
ESA         SYYCTCPR----SRIQQ--------LG-----------------GIYDGHCRAL-----------------------RHGPE-
NAAVRL
ENT         SYYCTCTR----ARIQS--------AG-----------------GVYDGHCRHL-----------------------NLGPE-
NAAVRL
DDA         SYHCTCTR----QRIQQ--------LG-----------------GFYDGHCRDL-----------------------AQPPY-
HTALRL
YPI         SYYCTCTR----SRIHQ--------LG-----------------GFYDGYCRDR-----------------------HLPAS-
GAAIRL
RAH         SYYCTCTR----QRIAQ--------IG-----------------GTYDGHCRSL-----------------------QLSPE-
NAAIRL
PAM         SYFCTCTR----SRIQQ--------AG-----------------GFYDGHCRDR-----------------------QRGPE-
GAALRL
EIC         AYYCTCTR----QRIHA--------LG-----------------GLYDGHCRYL-----------------------AQGAL-
GAALRL
PLU         SYYCTCTR----QRIHQ--------IN-----------------GFYDRNCREL-----------------------NLPID-
HAAIRF
XBO         SYYCTCTR----QRIQQ--------LG-----------------GFYDDHCRHL-----------------------HLLAD-
NAAVRL
DSH         LYPCSCSR----RDIQE--------AARAPQEGAP---LLGPDGVVYPGTCRGRDR--------------------GGARPE-
GAVLRL
RDE         TYPCTCSR----KDIQL--------AASAPQEGST--PVFGPDGLVYPGTCRAGSQ--------------------AVSGPP-
APAIRL
SIT         TYPCRCNR----ADIEA--------AAGAPQEG-V--PQFGPDGRIYPGTCRQRPL--------------------SDARPT-D-
VIRL
KVU         LYPCTCNR----RDIEA--------AASAPQEGV---PHFGPDGLIYPGTCRHKP---------------------
RPAAMPMDLPLRL
PDE         TYPCRCRR----ADIRA--------ALSAPQEGAA--PVIGPDGPVYPGTCRHRR---------------------MADAGP-
DDAIRL
CTT         AYPCGCTR----KDIEA--------AWLAQGLTHER-----HVERPYPGTCRHG-------LQGKPARA-----WRFALEQQ-
VHDLQE
POL         AYPCACTR----QDIAR--------AHAASGKARER-----HGELVYPGTCRQG-------LHGRPARA-----WRFLTETS-
QENTRL
MPT         AYPCVCTR----QQIVA--------ANSARGLLPSR-----HAELVYPGTCRPEN----GGLHDRRAR-----------------
SWRL
```

```
HHA         AYPCGCTR----KEIQA--------AASAHGPIG----------VVYPGTCRDG----------------------LPAGRA-
ERAVRV
BMA         VYPCGCSR----KEIAD--------SLRAAHERHT--------TLAYPGTCRTG-----------------------LHGKP-
ARAWRL
RSO         LYPCGCTR----REIAD--------SITTVDAQGRLR----HQTLIYPGTCRNG----------------------LMGRP-
PRAWRV
GLO         VYPCSCSR----AEIAR--------SASAP-HPGE--------EILYPGTCRNG----------------------LPAGRE-
PKAWRL
SAL         VYPCFCTR----ADIAA--------SLSAPHGPS---------GAIYPGTCRDL-------PAAERAR--------RRAA-E-
PHCWRL
SJP         LYPCFCTR----ADIAA--------SAAAPHGPE---------GPVYPGTCRRL-------DEAARAR--------RIAAGE-
PHAWRI
SWI         VYPCFCTRARIAAEIAA--------SAAAPHGPD---------GPLYPGLCREL-------GEDERAA--------RIAAGE-
AHAWRL
NAR         LYPCTCTR----AEIAA--------AAVA-QGPD---------GPLYPGTCLRR----------------------GPVPGP-
DVAWRL
MCH         IYPCFCSR----GQIAA--------EVAARQARGAPVSRDPDGAPLYPGTCRAL-------SADARRA--------RIGQGE-
PHTWRL
BID         IYPCFCSR----TDIMQ--------KIRERAFWARDP----DGSPLYPGTCKHL-------PKDKIQE--------RLEAGE-
PASYRL
MSL         AYPCFCSR----GEIMA--------AVACKPDWPRDP----DGSPLYPGLCKHL-------SPSERAR--------RLAGGR-
SAAMRI
AZL         LYPCFCTR----KDIAAEIARAAAAPHGPGPTSSD--------GPLYPGTCRHR-------SPQERAE--------RIGDGE-
SWALRL
RRU         LYRCFCTR----SEIAAEVARSPSAPHGPE-------------GAPYPGLCKRL-------DPAEVAD--------RLAKGD-
AYALRL
ACR         LYRCFCTR----AAIAAAL----GAPHGAE--------------AAYPGTCRGL-------DPALADA--------RAAQGE-
PYALRL
SUR         VYPCFCTR----AEIAR--------AASAPHGLSD-------EGPRYPGTCARL-------SLAERA---------ERSRTR-
PPAYRF
DVU         VYPCYCTR----KELRT--------LAGAPHPGDL--------GAPYPGTCRNL-------TIEECSR--------KEKEGR-
RPAMRL
AFW         VYPCFCSR----AEIAA--------SAQAPHGPSD-------DGPRYPGTCRAL-------SAAERA---------ERARTR-
APSWRF
ADE         AYPCFCSR----AEIAA--------ASQAPHGASD-------EGPRYPGTCREL-------SPAEVA---------RRSASR-
RPAWRL
ACA         IYPCTCSR----RDLLR--------AAQAPHEGEGGG----DEGPMYPGTCRPP-------RTLRSRPGME--EERRYRSPA-
GANWRF
ABA         VYPCTCSR----KDLAL--------SAQAPNEGDE--------EPVYPGKCRPA---------PGATF--------ETIDPA-
AVNWRF
JAN         LFPCDCSR----RDIQA--------ALSAPQETAS--PPLGPDGIIYPGTCRH--------RTPHPN--HADPGPMPD-
GVALRLRVDE
```

Group-II: *No ZB-motif*

```
ECO GluRS   AYKCYCSK----ERLEA--------LREEQMAKGE--------KPRYDGRCRHS-------HE-------------HHADDE-
PCVVRF
ECO         SYYCTCTR----ARIQS--------IG-----------------GIYDGHCRVL----------------------HHGPD-
NAAVRI DAK         LFFCHCTR----RQIKK--------ISADHLYPGTCRS--RFQAPVEPHTVRIR-------VPHRWVF-------
DPASQEA-AWPFAA
SCL         LYPCSATR----EEVRA-------IGRRAPDG-----------GFAYDNRARGR-------------------------ALP-
PGGWRA
AEX         VYACYKTR----KELAQ--------AAQGAPQ-----------------------------EGDAVAG--------DGPAER-
EPSWRL
BSB         LYRCFRTR----KELMA--------LAGVAPHADD-------PADAQAFAGGPL-------DPAEETE--------RLGAGQ-
PFAWRL
PZU         LYRCFRTR----KEVAQ--------EIARAPHGA-----------MEVFRGAPL-------PRGEEAR--------RLAAGE-
PFAWRL
CAK         VYRCFRTR----KAL----------DIGRAPHEP-----------AAPFTGAPL-------PPDDEAA--------RLDRGE-
AFAWRL
CCR         VYRCFKTR----KEI----------DIGRAPHEP-----------AIAYVGAPL-------GADEEAQ--------RLARGE-
PFAWRL
CSE         VYRCFKTR----KEI----------DIGRAPHEP-----------AVAYVGAAL-------PPEEEAQ--------RLARGE-
AFAWRL
PLA         VYPCFATR----KEIQD--------AIIASGVPLQNWPRDPDGALLYPGIYKDI-------PRAKLNK--------LMWEGR-
TYAWRL
OCA         AYPAFESR----AEIVR---------MIGQSPRKPWPR-DPDGAPLYPGSVASL-------SKEERQV--------RLQAGE-
AFVMRL
BJA         VYPAFESR----AEIAR--------LVAAREADGPWPR-DPDGAPLYPGDAKSL-------SAGEQAR--------LIASGA-
PYALRL
NHA         LYPSFESR----AGIAG--------LVKAREADGVWPR-DPDGAPLYPGAAKSL-------SATERER--------RLRSGA-
AYALRL
RPD         VYPAFESR----AEIAR--------QVAARQVAGPWPH-DPDGAPLYPGAGKQL-------DPDERER--------LIGSGA-
PYALRL
```

```
XAU        VYPAFESR----SEIAG--------AVIGQESATG-------RPAPRDPDGAPL-------NPFPRAAMSDAARAALRDQGA-
PFVVRL
AZC        LYASFETR----AEIAR--------AVLAREAAGR--------SWPRDPDGVPL-------YPFSRESMTEAARARRKAEGE-
PYVLRL
```

Supplementary Figure S3. ZB-motif-based classification and sequence alignment of pZBDs of bacterial Glu-Q-RSs (see Table S2 for abbreviations; *E. coli* GluRS and Glu-Q-RS (shown in green; pdb ID: 4a91) are shown in each group for reference where the Zn-binding amino acid residue are marked in red).

archaeal GluRS **Group-I: CxCx$_{14}$CxC**

Escherichia coli GluRS **AYKCYCSKERLEALREEQMA-----KGEKPRYDGRCRHS-------**
HEHHADDEPCVVRF
Methanothermobacter thermautotrophicus AYVCTCRPEEFRELKNRGEA-----CHCRSLGFRENLQR--WREMFEM---
KEGSAVVRV
Methanobrevibacter smithii AYMCTCDGATFKELKDNCKP-----CPCRDNSVEKNLEL--WDKFDQM---
HAGEAVLRV
Methanopyrus kandleri AYVCTCDPDEFRRLRDVGRA-----CPCRSRDKEENLEL--WEEMLDG-
TFSEGEAVVRV
Methanococcus maripaludis AYVCDCDADEFRTLREQGTL-----CKCRDTIPEENLKL--WEKMLAG---
ELDNVAVRL
Methanocaldococcus jannaschii AYVCDCNPEEFRELRNKGVP-----CKCRDRAIEDNLEL--WEKMLNG---
ELENVAVRL
Nitrosopumilus maritimus AYVCTCKREDISQNRRERKA-----CKCSMEDVGKNNKN--WEKMQNK--
FKPGEAIVRF
Cenarchaeum symbiosum A AYVCTCKRDDMSKNRRAMAP-----CKCSKKAQEDHMEG--WDKMHGK--
FKPGQAVARF

archaeal GluRS **Group-II: CxCx$_{14}$CxH**

Escherichia coli GluRS **AYKCYCSKERLEALREEQMA-----KGEKPRYDGRCRHS-------**
HEHHADDEPCVVRF
Methanothermobacter thermautotrophicus AYVCTCRPEEFRELKNRGEA-----CHCRSLGFRENLQR--WREMFEM---
KEGSAVVRV
Methanohalobium evestigatum AYVCFCKGDEFKKYKDNKKA-----CPHRNQSPEDNLYY--WNNMLNG-
EYGEKEAVLRI
Methanosarcina acetivorans AYVCFCKGEDFKRLKDAKQA-----CPHRDTSPEENLMH--WEKMLAG-
EYEDQQAVLRI
Archaeoglobus fulgidus AYTCFCSQEEFKKFRDSGEE-----CPHRNISVEDTLEV--WERMLEG-
DYEEGEVVLRI
Ferroglobus placidus AYVCFCEREEFKKYKDAGEE-----CPHRNTPAEDNLEF--WRKMLEG-
EYKEGEAVLRI
Ignicoccus hospitalis AYVCTCPPEEWRKLRDEGKP-----CPHRDLPPEEQEEL--LDQVLEG-
KFGEGEAVVRV
Thermococcus onnurineus AYVCTCPPEKFRELRDNGIA-----CPHREEPVEVQLER--WRKMLNG-
EYKEGEAVVRI
Pyrococcus horikoshii AYVCTCPPEKFRELRDKGIP-----CPHRDEPVEVQLER--WKKMLNG-
EYKEGEAVVRI
Natronomonas pharaonis AYTCSCDAESFSELKNNAEA-----CPHRDKDIETTLSE--FEAMIDG-
EYSAGEMVLRV
Halogeometricum borinquense AYTCSCSGEDFSELKNAGEP-----CPHRDKDPETTREE--FEAMIDG-
EYSSGEMVLRI
Natrialba magadii AYTCSCSGDEFSELKNSGEP-----CPHRDKDVETVRSE--FEDMVAG-
EYESGEMVLRV
Halalkalicoccus jeotgali AYTCTCPGETFSELKNSGEA-----CPHREKSVEESMDE--FEEMVAG-
EYNAGEIVLRV
Halorhabdus utahensis AYTCSCPGETFSELKNSGEA-----CPHREKDAATVHEE--FDAMVDG-
EYDPGEMVLRV
Haloarcula marismortui AYTCSCPQGEFSDLKNNGEA-----CPHRDKDAETTRSE--FEAMVDG-
EYDSGEMVLRV
Halobacterium sp. NRC-1 AYTCSCPGAEFSDLKNSGEA-----CPHRDKDPETVADE--FEAMVDG-
EYNSGEMVLRV
Haloquadratum walsbyi AYTCSCSGEHFSKLKNAGEA-----CPHREKSVNQTQTE--FEAMIHG-
EYSAGEMVLRV
Nanoarchaeum equitans AYVCLCKAEEFREYRNKGLP-----CPHRNQSIEENLEL--WSKMLKG-
EFKKGEAVVRL

archaeal GluRS **Group-III:** *No ZB-motif*

Escherichia coli GluRS **AYKCYCSKERLEALREEQMA-----KGEKPRYDGRCRHS-------**
HEHHADDEPCVVRF
Methanothermobacter thermautotrophicus AYVCTCRPEEFRELKNRGEA-----CHCRSLGFRENLQR--WREMFEM---
KEGSAVVRV
Thermofilum pendens AYVCTHSQDEIKAFRDAGKP-----DPCSFLPPEEHMER--WEKMLSG-
EYPEGAAVLRI
Methanohalophilus mahii AYVCFCEGADFKKYKDSCTP-----CPDRDRNPAENLEH--WHKMIEG-
AYEEKSAVLRI
Methanocorpusculum labreanum AYVCFCEGADFKKYKDSCTP-----CPDRDRNPAENLEH--WHKMIEG-
AYEEKSAVLRI
Picrophilus torridus MYIAETDQKRFKELKLKSMA-----LPDRSMDPGVHLDR--FDKMLNR-
EYREGEAVAL

```
Aciduliprofundum boonei             AYITTVPAEEWRKLKSEGKP-----TKDRELPPEEQLER--WEKMLAG-
EYDEGEAVYVV
Caldivirga maquilingensis           AYVVAKGSGCEPDDWKRLKIEG-KPCLTREAESSRNLEL--FDKMLEG-
AFNEGEAIVAV
Hyperthermus butylicus              AYVDDRSPEEFRRYRDEGRLE---DYPPRKRSVEENLEL--WDKMVSG-
AFGEGEAVLRV
Acidilobus saccharovorans           AYVDLGGEESKKLISE-GKP-----PEYRDKPPEWQLEQ--FDRMLSG-
HYKEGEAVVRF
Thermosphaera aggregans             AYIDKCGEKEFKAWRREGRA-----CPHRELPVETQLEE--FDKMLSG-
HYGEGEAVVRV
Staphylothermus hellenicus          AYVDKCGPKEFKKLRDAGKP-----CPHRELSPEKHLEE--LDKIFEG-
YYSEGEAVVRV
Desulfurococcus kamchatkensis       AYIDTCPDKEFRLYRNAGKA-----CPHRNTSVEENLEK--FDKMLEG-
HYGEGDAVLRV
Sulfolobus acidocaldarius           AYIDLCKEAEFKERRSKREA-----CPHRETSPESNLEL--FEKMIHG-
EFEEGKAVVRL
Metallosphaera sedula               AYVDTLSDAEFKAWRDSRNKT---VYKPRTNPPEVNLEL--WEKMLNG-
DFDEGKAVVRI
Sulfolobus islandicus               AYVDTCDSSTFKKFRDSRGKMREPECLHRSSSPESNLEL--FEKMLEG-
MFKEGEAVVRL
```

Supplementary Figure S4. ZB-motif-based classification and sequence alignment of pZBDs of archaeal GluRSs (see Table S3 for abbreviations; *E. coli* GluRS and *M. thermautotrophicus* GluRS (shown in green; pdb ID: 3aii) are shown in each group for reference where the Zn-binding amino acid residue are marked in red).

eukaryal GluRS **Group-I:** $CxCx_{20}Yx_3C$

Escherichia coli GluRS **AYKCYCSKERLEALREEQMA-----KGEKPRYDGRCRHS-------**
HEHHADDEPCVVRF
Dictyostelium discoideum AYHCFCSSERLSMSRINLKN-----QHAMSLYDRHCLKL--
SEDEIQRKLQSGESHTIRL
Bos taurus AYPCFCSPQRLELLKKEALR-----NRQTPRYDNRCRSL--
SQAQVAQKLATDPKPAIRF
Danio rerio AYYCFCSNQRLELLKKEAQR-----SGHAPRYDNRCRRL--
QPQQVEQKLAAGVPAVVRF
Aedes aegypti AYYCFCSERRLELLRKEAVR-----LRQVPKYDNKCRHL--
TPGQVAERLAKNDKFCIRF
Acyrthosiphon pisum AYKCYCTEKRLELLRRDALR-----TRTIPRYDNKCRSL--DDEDL--
SHNEGKKYCIRF
Schistosoma mansoni AYRCFCSSERLAILRKEQNR-----RREPQRYDNRCRNL--SQREIDEN-
LASLPYVIRF
Aspergillus fumigatus AYRCFCSPERLDSLARHRSQ-----AGLPPGYDRQCADI--
SAEESEDRAAKGEAHVVRL
Plasmodium yoelii AYYCFCTKDELNEKKEMTKA-----MKKKYIYDRKCRHL--
NDDTINKYLGENKLYAIRF
Plasmodium vivax AYFCFCTKDELSEKKEKAKL-----VRTKYTYDRACRDL--
SDEMVKQNLSKKKPYTIRF
Plasmodium falciparum AYFCFCSKEELQEIKEKSKM-----MKKKYIYNRKCRDM--
NNEQIKMKLEQNIAYTIRF
Babesia bovis AYRCFCSKEDVERRRKNSEE-----DNSQITYDKTCSHV--
TSEGVEVALKSGRKYTVRL

eukaryal GluRS **Group-II:** $CxCx_{20}Cx_3C$

Escherichia coli GluRS **AYKCYCSKERLEALREEQMA-----KGEKPRYDGRCRHS-------**
HEHHADDEPCVVRF
Brugia malayi AYRCFCCDDTWKEVDPVKKE-----RPKLLPCLKECFTK--
TRSESKSMVFDGMPHVVRL

eukaryal GluRS **Group-III:** ***No ZB-motif***

Escherichia coli GluRS **AYKCYCSKERLEALREEQMA-----KGEKPRYDGRCRHS-------**
HEHHADDEPCVVRF
Phaeodactylum tricornutum AYRCFCTPEELDAMKADQEA-----RGETPRYDGRWRDA--
SVDDVQQALDEGKAYTVRF
Ricinus communis VYRCFCSNEELEKMKEIAKL-----KQLPPVYSGKWATA--
TDEEVQEELAKATPYTYRF
Arabidopsis thaliana VYRCFCSSEELVKMKENAKL-----KQLPPVYTGKWATA--
SDAEIEQELEKGTPFTYRF
Trypanosoma brucei AYCDKTTREEMQKCRFNGVP-----TSYRDISIEETKRM--WSEMKEG-
SAEGQETCLRA
Leishmania major AYCDKTPREEMQKCRFDGIP-----TKYRDASVEENMRL--WREMKKG-
TKEGQITCLRA
Lodderomyces elongisporus AYCDDTPSDKMREERMVGDA-----SARRERSIEENLRV-FTEEMKNG-
TEEGLKNCLRA
Saccharomyces cerevisiae AYCDDTPTEKMREERMDGVA-----SARRDRSVEENLRI-FTEEMKNG-
TEEGLKNCVRA
Neurospora crassa AYADDTDQDTMRDQRWKGIA-----SARRDRSVEENLRI-FTEEMKNG-
TEEGLKNCIRA
Entamoeba histolytica AYIDDIDPEEMHKGRMEGIE-----SPNRNNSLEKNMKM--WEEMKNG-
TEYGKKCVMRA
Encephalitozoon cuniculi AYADNTPQEVMRDERGRGVE-----SRCRSMDVEESKRI--FKEMARG---
NASGYCLRA

eukaryal GluProRS ***No ZB-motif***

Escherichia coli GluRS **AYKCYCSKERLEALREEQMA-----KGEKPRYDGRCRHS-------**
HEHHADDEPCVVRF
Equus caballus AYVDDTPAEQMKAEREQRIE-----SKHRKNSIEKNLQM--WEEMKKG-
SQFGQSCCLRA
Xenopus laevis AYVDDTPAEQMKSEREQRIE-----SKHRSNTVAKNMEM--WEDMKKG-
TEYGQTCCLRA
Rattus norvegicus AYVDDTPADEMKAE-----------------PVEKNLQM--WEEMKRG-
SQFGQSCCLRA
Mus musculus AYVDDTPAEQMKAEREQRTE-----SKHRKNSVEKNLQM--WEEMKKG-
SQFGQSCCLRA
Pan troglodytes AYVDDTPAEQMKAEREQRIE-----SKHRKNPIEKNLQM--WEEMKKG-
SQFGQSCCLRA

```
Canis familiaris              AYVDDTPAEQMKAEREQRIE-----SKHRKNSVEKNLQM--WEEMKKG-
SQFGQSCCLRA
Sus scrofa                    AYVDDTPAEQMKAEREQRIE-----SKHRANSVEKNLQM--WEEMKKG-
SQFGQSCCLRA
Gallus gallus                 AYVDDTPAEQMKAEREQRME-----SKHRNNCVNKNLQM--WEEMKKG-
TEYGQTCCLRA
Culex quinquefasciatus        AYVDDTEPEQMKKERDERTE-----SKNRSNSVEKNLQM--WREMVKG-
SAAGQKCCVRA
Drosophila melanogaster       AYVDDTPPEQMKLEREQRVE-----SANRSNSVEKNLSL--WEEMVKG-
SEKGQNTACAA
Homo sapiens                  AYVDDTPAEQMKAEREQRID-----SKHRKNPIEKNLQM--WEEMKKG-
SQFGQSCCLRA
```

Supplementary Figure S5. ZB-motif-based classification and sequence alignment of *pZBD*s of eukaryal GluRS and GluProRS (see Table S4 for abbreviations; *E. coli* GluRS is shown in each group for reference where the Zn-binding amino acid residues are marked in red).

A

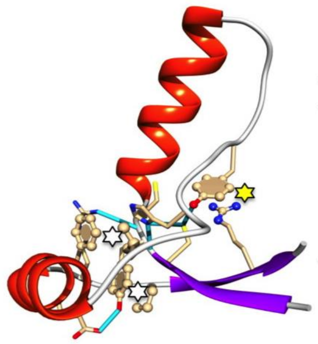

Key H-bonds		
Donor	Acceptor	D-A(Å)
CYS98 SG	HIS125 ND1	3.85
CYS98 SG	TYR121 OH	3.07
TYR99 N	HIS125 ND1	2.89
TYR99 OH	GLU137 OE2	2.70
ARG141 NH1	TYR99 O	2.87

T. elongatus GluRS (2cfo)

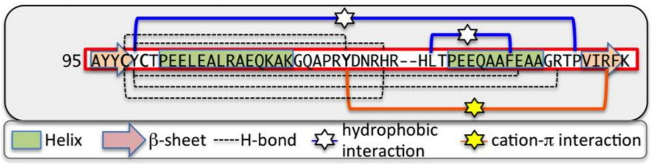

B

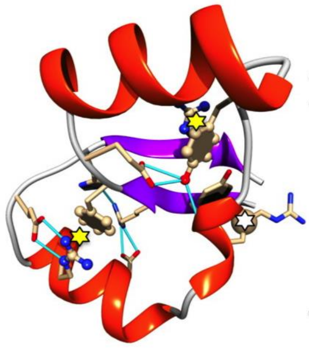

Key H-bonds		
Donor	Acceptor	D-A(Å)
TYR122 OH	GLU107 OE1	2.69
TYR122 OH	GLU107 OE2	3.30
ALA126 N	TYR122 OH	2.92
ARG137 NH2	GLU142 OE2	2.64
ARG137 NE	GLU142 OE1	2.91
ARG104 NH1	GLU135 OE1	3.49
ARG104 NH1	GLU135 OE2	2.97
ARG104 NE	GLU135 OE1	2.70

T. thermophilus GluRS (2cuz)

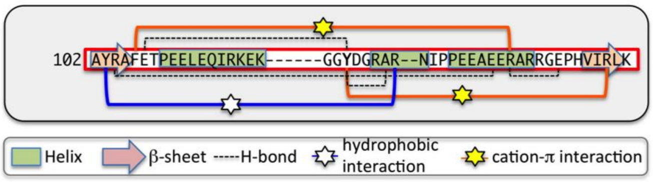

(Continued in next page)

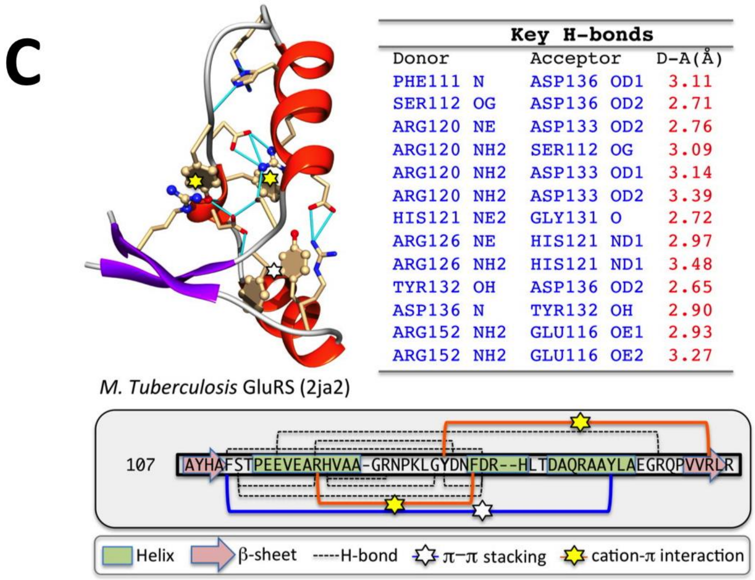

Key H-bonds

Donor	Acceptor	D-A(Å)
PHE111 N	ASP136 OD1	3.11
SER112 OG	ASP136 OD2	2.71
ARG120 NE	ASP133 OD2	2.76
ARG120 NH2	SER112 OG	3.09
ARG120 NH2	ASP133 OD1	3.14
ARG120 NH2	ASP133 OD2	3.39
HIS121 NE2	GLY131 O	2.72
ARG126 NE	HIS121 ND1	2.97
ARG126 NH2	HIS121 ND1	3.48
TYR132 OH	ASP136 OD2	2.65
ASP136 N	TYR132 OH	2.90
ARG152 NH2	GLU116 OE1	2.93
ARG152 NH2	GLU116 OE2	3.27

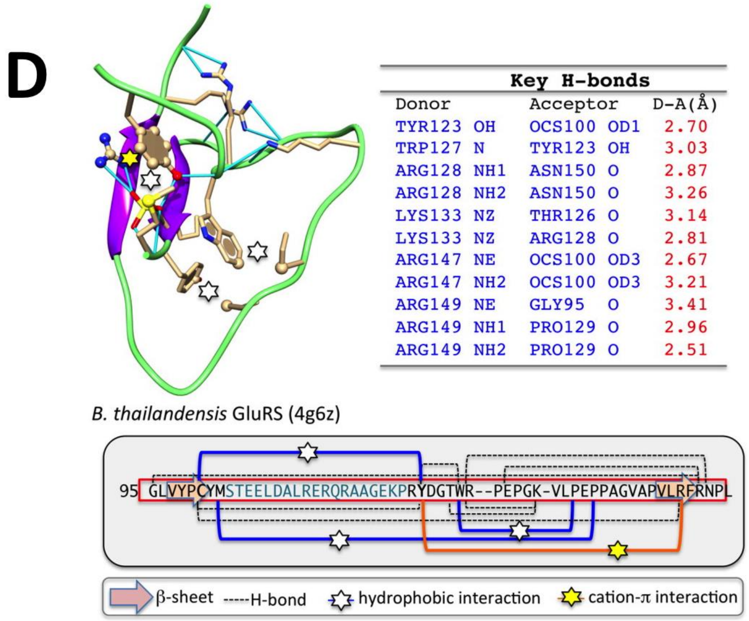

Key H-bonds

Donor	Acceptor	D-A(Å)
TYR123 OH	OCS100 OD1	2.70
TRP127 N	TYR123 OH	3.03
ARG128 NH1	ASN150 O	2.87
ARG128 NH2	ASN150 O	3.26
LYS133 NZ	THR126 O	3.14
LYS133 NZ	ARG128 O	2.81
ARG147 NE	OCS100 OD3	2.67
ARG147 NH2	OCS100 OD3	3.21
ARG149 NE	GLY95 O	3.41
ARG149 NH1	PRO129 O	2.96
ARG149 NH2	PRO129 O	2.51

Supplementary Figure S6. Intra-chain interactions in *pZBD*s without a bound Zn^{2+} ion.

(**A**) *pZBD* of *T. elongatus* GluRS. (**B**) *pZBD* of *T. thermophilus* GluRS. (**C**) *pZBD* of *M. tuberculosis* GluRS. *pZBD* of *B. thailandensis* GluRS.

A

		12345678901234567--890ab1234567890123-456-78901234567---890123456789012345678901 23
pZBD-deleted	>ACL_E1A_TE	GGCCCGUUGGAGAAAC---GGUU-AACUCACAUGCCU-UUC-ACGCAUGCACU---CACGGGUUCGAAUCCCGUACGGGUCA
	>ACL_E1B_TE	GGCCCGUUGGAGAAAC---GGUU-AACUCACAUGCCU-UUC-ACGCAUGCAUU---CACGGGUUCGAAUCCCGUACGGGUCA
	>PML_E10_TE	GGCCCGUUGGAGAAAC---GGUU-AACUCACAUGCCU-UUC-ACGCAUGCAUU---CACGGGUUCGAAUCCCGUACGGGUCA
	>POY_E10_TE	GGCCCGUUGGAGAAAC---GGUU-AACUCACAUGCCU-UUC-ACGCAUGCAUU---CACGGGUUCAAAUCCCGUACGGGUCA
pZBD-containing	>MFL_E10_TE_CCYC	GGCCUGUUGGUGAAGC---GGUU-AACACACACGGUU-UUC-AUCCGUGGACA---CACGGGUUCGAACCCCGUACAGGCUA
	>MPN_E10_TE_CCYC	GGCGCGUUGGUGAAGAG--ACUU-AACACACACGCCU-UUC-ACGCGUGCAUU---CACGGGUUUGAAUCCCGUACGCGUCA
	>UUE_E10_TE_CCYC	GGCCUGUUGGUGAAGUG--ACUG-AACACACACGCCU-UUC-ACGCGUGCAUU---CACGGGUUUGAAUCCCGUACAGGUCA
	>LMF_E10_FI_CCYC	GGCCCGUUGGUCAAGC---GGUU-AAGACACCGCCCU-UUC-ACGGCGGUAA----CACGGGUUCGAAUCCCGUACGGGUCA
	>EFA_E10_FI_CCYC	GGCCCGUUGGUCAAGC---GGUU-AAGACACCGCCCU-UUC-ACGGCGGUAA----CACGGGUUCGAGUCCCGUACGGGUCA
	>BSS_E1A_FI_CCYH	GGCCCGUUGGUCAAGC---GGUU-AAGACACCGCCCU-UUC-ACGGCGGUAA----CACGGGUUCGAAUCCCGUACGGGUCA
	>BSS_E1B_FI_CCYH	GGCCCGUUGGUAAAC----GGU--UAAGCACCGCCCU-UUC-AC-GCGGUAA----CACGGGUUCGAAUCCCGUACGGGUCA

B

		12345678901234567--890ab1234567890123-456-78901234567---890123456789012345678901 23
pZBD-deleted	>MIN_E20_VE	GGCCCCAUCAUCUAGUU--GGUU-AGGAUACGGCCCU-CUC-AAGGCCGGUG----GACGGGUUCGAAUCCCGUUGGGGCUA
	>MIN_E10_VE	GACCCCAUGGUGUAGU---GGUC-AGCACUCCACCCU-UUC-ACGGUGGCAG----CACGGGUUCGAAUCCCGUUGGGGUCG
	>AMU_E10_VE	GUCGCGUUCGUCUAGC---GGUCCAGGACUCCCGCCU-UUC-ACGCGGGCAA----CACGGGUUCGAGUCCCGUACGCGAUG
	>PHM_E10_PL	GCUCCCGUCGUCUAGUCA-GGUCUAGGACACCUGGUU-UUC-AUCCAGGCGA----CACGGGUUCGAAUCCCGUCGGGAGUA
	>PHM_E20_PL	GCCCCCUUCGUCUAGU---GGCCUAGGACAUCGCCUU-CUC-AUGGCGAGAA----CAGGGGUUCGACUCCCCUAGGGGGUA
pZBD-containing	>RBA_E1C_PL	GGCGUGGUAGAUUCUGAU-GGCA-GAAUCGUCUGGUU-UUC-AUCCAGGAGU----CCGCGGGUUCGAUUCCCGCUCACGUC
	>RBA_E1B_PL	GGCGUGGUAGAUUCUGUU-GGCA-GAAUCGUCUGGUU-UUC-AUCCAGGAGUC---CGCGGGUUCGAUUCCCGCUCACGUCA
	>RBA_E1A_PL	GGCCCCUUCGUCUAGC---GGUCUAGGACAUCGCCCU-UUC-ACGGCGGUAA----CACGGGUUCGAGUCCCGUAGGGGUCA
	>RBA_E20_PL	GCCGCAUUCGACUACU---GGCU-AGGUCGGCUGCUU-CUC-AGGCAGCAGG----AAGGAGAUCGAAACUCCUAUGCGGUA
	>PSL_E10_PL	GGCCCCAUCGUCUAGA---GGCCUAGGACAUCGCCCU-UUC-ACGGCGGUAA----CAGGGGUUCGAAUCCCCUUGGGGUCA
	>PSL_E20_PL	GCUCCCUUCGUCUAGA---GGCCUAGGACCCCGGGUU-CUC-AGUCCGGUAA----CACGGGUUCGACUCCCGUAGGGAGUA
	>PLM_E10_PL	GGCCCCAUCGUCUAGA---GGCCUAGGACAUUGGAUU-UUC-AGUCCAAGAA----CUGGGGUUCGAAUCCCCAUGGGGUCA
	>IPA_E10_PL	GGCCCCGUCGUCUAGCCA-GGUCCAGGACACAGGAUU-UUC-AUUCCUGCAA----CACGGGUUCGAAUCCCGUCGGGGUCA
	>IPA_E20_PL	GCCCCCAUCGUCUAGU---GGCCUAGGACACUGGAUU-CUC-AGUCCAGGAA----CCGGGGUUCGAGUCCCCGUGGGGGUG

Supplementary Figure S7. tRNA^{Glu} sequence comparison between bacteria with and without *pZBD*s in their GluRSs.

(**A**) Comparison of three tRNA^{Glu} sequences of tenericutes with *pZBD*-deleted GluRS and tenericutes/firmicutes with *pZBD*-containing GluRS. These two groups appear as sister clades in GluRS phylogenetic tree. Nucleotides at 11-24 are different in the two groups. (**B**) Comparison of tRNA^{Glu} sequences of one planctomycetes and two verrucomicrobia (with *pZBD*-deleted GluRS) and planctomycetes with *pZBD*-containing GluRS. These two groups appear as sister clades in GluRS phylogenetic tree.

A

		GlnRS/gatB		12345678901234567--890ab1234567890123-456-78901234567---89012345678901234567890123
pZBD-deleted	PML(te)	+/−	**tRNA-Gln1**	AGGCCCAUAGCCAAGU---GGU--AAGGCAACGGACU-UUG-ACUCCGUGAUU---CGUUGGUUCAAAUCCAGCUGGGCCUG
	ACL(te)	+/−	**tRNA-Gln1**	AGGCCCAUAGCCAAGC---GGU--AAGGCAACGGACU-UUG-ACUCCGUCACU---CGUAGGUUCAAAUCCUGCUGGGCCUG
	POY(te)	+/−	**tRNA-Gln1**	AGGCCCAUAGCCAAGU---GGU--AAGGCAACGGACU-UUG-ACUCCGUGAUU---CGUUGGUUCAAAUCCAGCUGGGUCUG
pZBD-containing	MFL(te)	−/+	**tRNA-Gln1**	UGGGCUAUAGCCAAGC---GGU--AAGGCAAGGGACU-UUG-ACUCCCUCAUG---CGCCGGUUCGAAUCCUGCUAGCCCAA
	MPN(te)	−/+	**tRNA-Gln1**	UGGGAUGUAGCCAAGC---GGU--AAGGCAAUAGACU-UUG-ACUCUAUCAUG---CGAUGGUUCGAUCCCAUCCAUCCCAG
	UUE(te)	−/+	**tRNA-Gln1**	UGGCCUGUAGCCAAGC---GGU--AAGGCAGAAGAUU-UUG-AUUCUUCCACG---CGUAGGUUCGAUCCCUACCAGGCCAG
	LMF(fi)	−/+	**tRNA-Gln1A**	UGGGCUAUAGCCAAGC---GGU--AAGGCAACGGAUU-UUG-AUUCCGUCAUG---CGCUGGUUCGAAUCCAGCUAGCCCAG
	LMF(fi)	−/+	**tRNA-Gln1B**	UGGGCCAUAGCCAAGC---GGU--AAGGCAACGGACU-UUG-ACUCCGUCAUG---CGUUGGUUCGAAUCCAGCUGGCCCAG
	MFL(fi)	−/+	**tRNA-Gln1**	UGGGGUAUAGCCAAGC---GGU--AAGGCAACGGACU-UUG-ACUCCGUCAUG---CGUUGGUUCGAAUCCAGCUACCCCAG
	BSS(fi)	−/+	**tRNA-Gln1A**	UGGGCUAUAGCCAAGC---GGU--AAGGCAACGGACU-UUG-ACUCCGUCAUG---CGUUGGUUCGAAUCCAGCUAGCCCAG
	BSS(fi)	−/+	**tRNA-Gln1B**	UGGGCUAUAGCCAAGC---GGU--AAGGCAAUGGACU-UUG-ACUCCGUGAU----CGUUGGUUCGAAUCCAGCUAGCCCAG
	OOE(fi)	−/+	**tRNA-Gln1**	UGCCCCAUAGCCAAGUC--GGU--AAGGCAGCGGUUU-UUG-GUACCGCCACG---CGCUGGUUCGAGUCCAGCUGGGGCAA

B

		GlnRS/gatB		12345678901234567--890ab1234567890123-456-78901234567---89012345678901234567890123
pZBD-deleted	PHM(pl)	+/+	**tRNA-Gln1**	AGGCCCAUGGUGUAAU---GGC--AGCACAGCUGUUU-UUG-GUGCAGCUCG----UCGGGGUUCGAAUCCCUGUGGGCCUA
	PHM(pl)	+/+	**tRNA-Gln2**	AGGCCCAUGGUGUAAC---GGU--AGCACUAAAGAUU-CUG-AUUCUUUCAG----UCGGGGUUCGAAUCCCUGUGGGCCUA
	AMU(ve)	+/+	**tRNA-Gln1**	AGCCCCAUAGUGUAAUU--GGU--AACACACCUGAUU-UUG-GUUCAGUAUU----UCUAGGUUCGAGUCCUGGUGGGGCUG
	MIN(ve)	−/+	**tRNA-Gln1**	UGCCCGGUGGUGUAAU---GGU--AGCACAGAGGUCU-UUG-GAGCCUUUAG----UCAUGGUUCGAAUCCAUGCCGGGCAG
	MIN(ve)	−/+	**tRNA-Gln2**	UGCCGCGUGGUGCAAUU--GGU--AGCACAUCAGACU-CUG-GAUCUGAGUGU---UCUAGGUUCGAAUCCUAGCGCGGCAG
pZBD-deleted	RBA(pl)	−/+	**tRNA-Gln1**	UACCCCGUGGUGUAAUU--GGC--AACACAGCUGGUU-UUG-GUCCAGCCAU----UCUAGGUUCGAGUCCUAGCGGGGUAG
	RBA(pl)	−/+	**tRNA-Gln2A**	GCCGGAGUAGCAGUUGUU-GGUC-GCUGCACCUGACU-CUG-AAUCAGGAGUU---CAUUGGUUCGAUUCCAAUCUCCGGUG
	RBA(pl)	−/+	**tRNA-Gln2B**	GCCGGAGUAGCAGUUGUU-GGUC-GCUGCACCUCGCU-CUG-AACGAGGAGUCCAUUGGUUCAAUUCCUGUCCUCGGCCCUG
	PSL(pl)	−/+	**tRNA-Gln1**	UCGGGGAUGGUGUAAU---GGU--AACACUACUGAUU-UUG-GUUCAGUCAU----UCUAGGUUCGAAUCCUAGUCCCCGAA
	PSL(pl)	−/+	**tRNA-Gln2**	UGACCUCUGGUGUAAC---GGU--AGCACGAAUGAUU-CUG-GAUCAUUUAG----UCUAGGUUCAAAUCCUAGGAGGUCAA
	PLM(pl)	−/+	**tRNA-Gln1A**	UCGGAUGUCGGCUAAU---GGU--AAGCCAACUGCCU-UUG-AAGCAGUGGUA---UGAAGGUUCAACUCCUUCCGCCCGGG
	PLM(pl)	−/+	**tRNA-Gln1B**	UACCCCGUAGUGUAAC---GGU--AGCACAGCAGGUU-UUG-GUCCUGCACG----UUCAGGUUCGAAUCCUGGCGGGGUAA
	PLM(pl)	−/+	**tRNA-Gln2**	UGCCUGAUGGUGUAAU---GGU--AGCACGAGUGACU-CUG-GAUCACUUAG----UUGAGGUUCGAAUCCUCAUCGGGCAA
	IPA(pl)	−/+	**tRNA-Gln1**	UACCCGGUGGUGUAAC---GGU--AACACAAGAGGUU-UUG-GUCCUCUCGU----UCUAGGUUCGAAUCCUAGCCGGGUAG
	IPA(pl)	−/+	**tRNA-Gln2**	UGGGGAAUGGUGUAAC---GGU--AGCACGCUUGACU-CUG-GAUCAAGUAG----UCUAGGUUCGAAUCCUAGUUCCCCAG

Supplementary Figure S8. tRNA^{Gln} sequence comparison between bacteria with and without *pZBD*s in their GluRSs.

(**A**) Comparison of three tRNA^{Gln} sequences of tenericutes with *pZBD*-deleted GluRS and tenericutes/firmicutes with *pZBD*-containing GluRS. These two groups appear as sister clades in GluRS phylogenetic tree. (**B**) Comparison of tRNA^{Gln} sequences of one planctomycetes and two verrucomicrobia (with *pZBD*-deleted GluRS) and planctomycetes with *pZBD*-containing GluRS. These two groups appear as sister clades in GluRS phylogenetic tree. The genomic status of GlnRS and gatB are also shown.

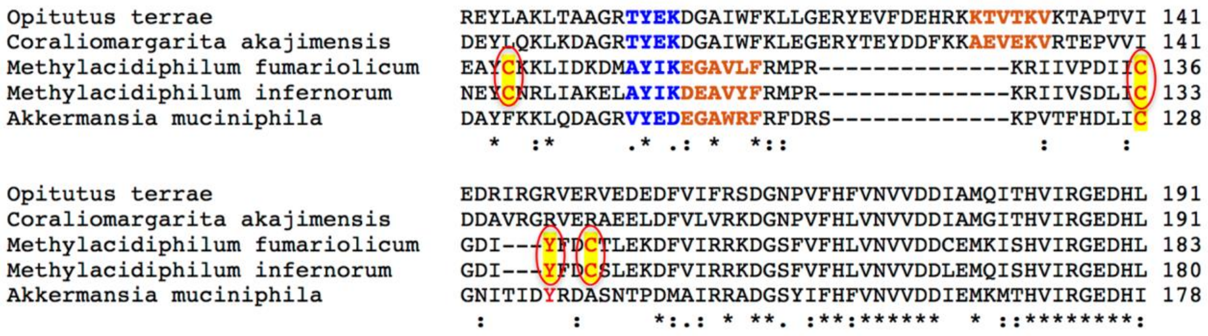

Supplementary Figure S9. Multiple sequence alignment of verrucomicrobial GluRS.

The *pZBD* and surrounding sequences of five verrucomicrobial GluRS sequences. Two β-sheets, flanking the *pZBD*, are colored blue and orange. Except *M. fumariolicum* and *M. infernorum*, all species contain GlnRS. Interestingly, these two bacteria show potential Zn-binding residues (marked yellow and highlighted by red ovals) in the neighborhood of *pZBD* that may lead to some unusual structural changes in this region.

Supplementary Table S1. NCBI-GI codes of 212 Bacterial GluRS used in this work.

Name of species	KEGG code	genera/phyla*	NCBI-GI
Escherichia coli	ECO	ga	16130330
Actinobacillus pleuropneumoniae	APL	ga	126208741
Francisella tularensis	FTT	ga	379725333
Haemophilus ducreyi	HDU	ga	33151555
Mannheimia succiniciproducens	MSU	ga	52424545
Pasteurella multocida	PMU	ga	15602980
Aeromonas hydrophila	AHA	ga	117620491
Idiomarina loihiensis	ILO	ga	56460871
Photorhabdus luminescens	PLU	ga	37525357
Oligotropha carboxidovorans	OCA	al	209885100
Bradyrhizobium japonicum	BJA	al	27379949
Nitrobacter hamburgensis	NHA	al	92117256
Rhodopseudomonas palustris	RPD	al	91977312
Pelobacter carbinolicus	PCA	de	404493274
Geobacter lovleyi	GLO	de	189423654
Desulfurivibrio alkaliphilus	DAK	de	297568488
Syntrophus aciditrophicus	SAT	de	85858037
Desulfobacca acetoxidans	DAO	de	328953376
Desulfarculus baarsii	DBR	de	302341545
Syntrophobacter fumaroxidans	SFU	de	116748805
Candidatus Desulfococcus oleovorans	DOL	de	158522544
Desulfobacterium autotrophicum	DAT	de	224370217
Desulfauibacillum alkenivorans	DAL	de	218782123
Hippea maritima	HMR	de	327398726
Rhodoferax ferrireducens	RFR	be	89901457
Cupriavidus metallidurans	RME	be	94311078
Bordetella bronchiseptica	BBR	be	33603345
Methylobacillus flagellatus	MFA	be	91775943
Thiobacillus denitrificans	TBD	be	74317752
Dechloromonas aromatica	DAR	be	71906954
Nitrosospira multiformis	NMU	be	82702731
Ralstonia solanacearum	RSO	be	17545898
Neisseria meningitidis	NMC	be	121635806
Laribacter hongkongensis	LHK	be	226940769
Desulfovibrio vulgaris	DVU	de	46580956
Lawsonia intracellularis	LIP	de	94986905
Desulfohalobium retbaense	DRT	de	258405532
Phenylobacterium zucineum	PZU	al	197105227
Caulobacter crescentus	CCR	al	16126148
Helicobacter pylori	HPY2	ep	15645267
Wolinella succinogenes	WSU2	ep	34556712
Campylobacter jejuni	CJR2	ep	57237686
Arcobacter nitrofigilis	ANT2	ep	296273255
Nitratiruptor sp.	NIS1	ep	152991382
Campylobacter jejuni	CJR1	ep	57238338
Helicobacter pylori	HPY1	ep	15645104
Wolinella succinogenes	WSU1	ep	34558490

Arcobacter nitrofigilis	ANT1	ep	296271698
Sulfurimonas autotrophica	SUA1	ep	307721946
Sulfuricurvum kujiense	SKU1	ep	313683490
Sulfurospirillum deleyianum	SDL1	ep	268680812
Sulfurovum sp.	SUN1	ep	152991704
Nitratifractor salsuginis	NSA1	ep	319957716
Acidithiobacillus ferrooxidans	AFE2	ga	198283983
Alkalilimnicola ehrlichei	AEH2	ga	114320962
Thioalkalivibrio sp	TGR2	ga	220934751
Halorhodospira halophila	HHA2	ga	121996917
Nitrosococcus oceani	NOC2	ga	77165713
Coxiella burnetii	CBU2	ga	215919194
Methylococcus capsulatus	MCA2	ga	53805249
Thioalkalivibrio sp	TGR1	ga	220934111
Acidithiobacillus ferrooxidans	AFE1	ga	198282724
Halorhodospira halophila	HHA1	ga	121998064
Alkalilimnicola ehrlichei	AEH1	ga	114320458
Nitrosococcus oceani	NOC1	ga	77163799
Coxiella burnetii	CBU1	ga	215918912
Methylococcus capsulatus	MCA1	ga	53804443
Ochrobactrum anthropi	OAN2	al	153009428
Brucella abortus	BMC2	al	189024181
Maricaulis maris	MMR2	al	114569537
Mesorhizobium loti	MLO2	al	13470967
Rhodospirillum rubrum	RRU2	al	83592027
Rhodobacter sphaeroides	RSP2	al	77464373
Paracoccus denitrificans	PDE2	al	119383635
Novosphingobium aromaticivorans	NAR2	al	87198989
Ehrlichia chaffeensis	ECH2	al	88658242
Orientia tsutsugamushi Boryong	OTS2	al	148285057
Neorickettsia sennetsu	NSE2	al	88608755
Rickettsia prowazekii	RPR2	al	15604193
Ochrobactrum anthropi	OAN1	al	153009373
Brucella abortus	BMC1	al	189024300
Paracoccus denitrificans	PDE1	al	119386425
Rhodobacter sphaeroides	RSP1	al	77462542
Mesorhizobium loti	MLO1	al	13470828
Ehrlichia chaffeensis	ECH1	al	88658087
Orientia tsutsugamushi Boryong	OTS1	al	148284334
Rhodospirillum rubrum	RRU1	al	83592936
Maricaulis maris	MMR1	al	114569946
Novosphingobium aromaticivorans	NAR1	al	87200047
Neorickettsia sennetsu	NSE1	al	88608680
Rickettsia prowazekii	RPR1	al	15604472
Mycoplasma pneumoniae	MPN	te	13508417
Ureaplasma urealyticum serovar	UUE	te	209554403
Bacteroides fragilis	BFR	ba	53715491
Porphyromonas gingivalis	PGI	ba	34541222
Parabacteroides distasonis	PDI	ba	150008894
Capnocytophaga ochracea	COC	ba	256820853

Gramella forsetii	GFO	ba	120437626
Flavobacterium johnsoniae	FJO	ba	146298767
Riemerella anatipestifer	RAN	ba	313206336
Salinibacter ruber	SRU	ba	83816484
Rhodococcus erythropolis	RER	ac	226305891
Corynebacterium glutamicum	CGL	ac	19552513
Renibacterium salmoninarum	RSA	ac	163839810
Micrococcus luteus	MLU	ac	239917386
Bifidobacterium adolescentis	BAD	ac	119025194
Isosphaera pallida	IPA	pl	320104035
Planctomyces limnophilus	PLM	pl	296122903
Pirellula staleyi	PSL	pl	283777822
Mycobacterium tuberculosis	MTU	ac	57117041
Streptomyces coelicolor	SCO	ac	21223902
Geodermatophilus obscurus	GOB	ac	284992441
Rhodopirellula baltica	RBA	pl	32474919
Methylacidiphilum infernorum	MIN	ve	189218195
Akkermansia muciniphila	AMU	ve	187736371
Brachyspira pilosicoli	BPJ	sp	404476278
Thermovibrio ammonificans	TAM	ht	319789482
Desulfurobacterium thermolithotrophum	DTE	ht	325295089
Leptospira interrogans serovar	LIE	sp	386075769
Opitutus terrae	OTE	ve	182414461
Coraliomargarita akajimensis	CAA	ve	294054239
Phytoplasma OY	POY	te	39938626
Candidatus Phytoplasma mali	PML	te	194246728
Bacillus subtilis	BSS	fi	305672791
Enterococcus faecalis	EFA	fi	29374701
Listeria monocytogenes	LMF	fi	46906470
Deferribacter desulfuricans	DDF	ht	291279461
Mesoplasma florum	MFL	te	50365466
Acholeplasma laidlawii	ACL	te	162447009
Oenococcus oeni	OOE	fi	116490418
Chlorobium limicola	CLI	gs	189345902
Ignavibacterium album	IAL	gs	385810346
Chlorobaculum tepidum	CTE	gs	21673138
Pelodictyon luteolum	PLT	gs	78187575
Prosthecochloris aestuarii	PAA	gs	194334583
Spirochaeta smaragdinae	SSM	sp	302339641
Thermus thermophilus	TTH	dt	46198378
Deinococcus radiodurans	DRA	dt	15805512
Meiothermus ruber	MRB	dt	291296951
Truepera radiovictrix	TRA	dt	297623515
Oceanithermus profundus	OPR	dt	313679650
Marinithermus hydrothermalis	MHD	dt	328950294
Leptotrichia buccalis	LBA	fu	257126047
Haliangium ochraceum	HOH	de	262197837
Fusobacterium nucleatum	FNU	fu	19704675
Sebaldella termitidis	STR	fu	269121949
Streptobacillus moniliformis	SMF	fu	269122997

Clostridium thermocellum	CTH	fi	125973166
Candidatus Sulcia muelleri	SMG	ba	502432114
Thermosynechococcus elongatus	TEL	cy	22298049
Microcystis aeruginosa	MAR	cy	166368010
Prochlorococcus marinus	PMC	cy	123965772
Synechococcus elongatus	SYF	cy	81301202
Cyanothece sp.	CYT	cy	172036673
Nostoc punctiforme	NPU	cy	186681815
Dictyoglomus thermophilum	DTH	ht	206902005
Aquifex aeolicus	AAE	ht	15606455
Thermocrinis albus	TAL	ht	289549304
Anabaena azollae	NAZ	cy	298491772
Phycisphaera mikurensis	PHM	pl	383765174
Alkaliphilus oremlandii	AOE	fi	158319518
Finegoldia magna	FMA	fi	169824111
Thermodesulfatator indicus	TID	ht	337286680
Candidatus Nitrospira defluvii	NDE	ht	302039291
Roseiflexus castenholzii	RCA	ns	156742292
Chloroflexus aggregans	CAG	ns	219849156
Dehalococcoides ethenogenes	DET	ns	57233878
Dehalogenimonas lykanthroporepellens	DLY	ns	300087234
Anaerolinea thermophila	ATM	ns	320160387
Chlamydia trachomatis	CTR	ch	15605172
Chlamydophila pneumoniae	CPA	ch	16752479
Parachlamydia acanthamoebae	PUV	ch	338174354
Candidatus Protochlamydia amoebophila	PCU	ch	46445878
Waddlia chondrophila	WCH	ch	297621898
Simkania negevensis	SNG	ch	338734090
Marinobacter aquaeolei	MAQ	ga	120554808
Gamma proteobacterium	GPB	ga	304312836
Azotobacter vinelandii	AVN	ga	226944119
Acinetobacter sp.	ACI	ga	50086349
Moraxella catarrhalis	MCT	ga	296113991
Hahella chejuensis	HCH	ga	83644930
Pseudomonas aeruginosa	PAE	ga	15598330
Teredinibacter turnerae	TTU	ga	254786566
Sinorhizobium medicae	SMD	al	150397997
Agrobacterium tumefaciens	ATU	al	159186179
Rhizobium etli	RET	al	86359246
Candidatus Liberibacter asiaticus	LAS	al	255764513
Sinorhizobium fredii	SFD	al	398355191
Pelagibacter ubique	PUB	al	71083617
Alpha proteobacterium	APM	al	406706166
Asticcacaulis excentricus	AEX	al	315497614
Bdellovibrio bacteriovorus	BBA	de	42523768
Myxococcus xanthus	MXA	de	108760344
Stigmatella aurantiaca	SUR	de	310820671
Anaeromyxobacter dehalogenans	ADE	de	86159019
Anaeromyxobacter sp	AFW	de	153004131

Desulfotalea psychrophila	DPS	de	51246589
Desulfobulbus propionicus	DPR	de	320354576
Koribacter versatilis	ABA1	ad	94967215
Acidobacterium capsulatum	ACA2	ad	225874464
Acidobacterium capsulatum	ACA1	ad	225873235
Koribacter versatilis	ABA2	ad	94967082
Thermosipho africanus	TAF2	ht	217076407
Fervidobacterium nodosum	FNO2	ht	154249365
Thermotoga maritima	TMA2	ht	15644103
Petrotoga mobilis	PMO2	ht	160903261
Kosmotoga olearia	KOL2	ht	239618313
Kosmotoga olearia	KOL1	ht	239618082
Petrotoga mobilis	PMO1	ht	160902068
Fervidobacterium nodosum	FNO1	ht	154249462
Thermosipho africanus	TAF1	ht	217077624
Thermotoga maritima	TMA1	ht	15644618

* The abbreviations stand for bacterial phyla and classes: α-proteobacteria (al), β-proteobacteria (be), δ-proteobacteria (de), ε-proteobacteria (ep), γ-proteobacteria (ga), hyperthermophilic bacteria (ht), acidobacteria (ad), spirochaetes (sp), bacteroidetes (ba), chlamydiae (ch), fusobacteria (fu), deinococcus-thermus (dt), (ns), green sulphur bacteria (gs), planctomycetes (pl), verrucomicrobia (ve), cyanobacteria (cy), actinobacteria (ac), firmicutes (fi), tenericutes (te).

Supplementary Table S2. NCBI-GI codes of 61 Bacterial Glu-Q-RS used in this work.

Name of species	KEGG code	NCBI-GI
Escherichia coli	ECO	226524698
Methylococcus capsulatus	MCA	53805241
Psychromonas ingrahamii	PIN	119944356
Klebsiella pneumoniae	KPN	152968725
Serratia proteamaculans	SPE	157372220
Sodalis glossinidius	SGL	85058469
Shigella dysenteriae	SDY	161950165
Salmonella enterica	STT	29140734
Cronobacter sakazakii	ESA	156935338
Enterobacter sp.	ENT	162286714
Dickeya dadantii	DDA	242240238
Yersinia pseudotuberculosis	YPI	153950577
Rahnella sp.	RAH	322834353
Pantoea ananatis	PAM	291616322
Edwardsiella ictaluri	EIC	238918731
Photorhabdus luminescens	PLU	37524870
Xenorhabdus bovienii	XBO	290476645
Dinoroseobacter shibae	DSH	159044023
Roseobacter denitrificans	RDE	110679837
Ruegeria sp.	SIT	99081234
Ketogulonicigenium vulgare	KVU	310816014
Paracoccus denitrificans	PDE	119386786
Comamonas testosteroni	CTT	264678533
Polaromonas sp.	POL	91788764
Methylibium petroleiphilum	MPT	124268084
Halorhodospira halophila	HHA	121998661
Burkholderia mallei	BMA	53725489
Ralstonia solanacearum	RSO	17546929
Geobacter lovleyi	GLO	189424013
Sphingopyxis alaskensis	SAL	103488560
Sphingobium japonicum	SJP	294010781
Sphingomonas wittichii	SWI	148556015
Novosphingobium aromaticivorans	NAR	87198575
Methylobacterium extorquens	MCH	218529152
Beijerinckia indica	BID	182677429
Methylocella silvestris	MSL	217979408
Azospirillum sp.	AZL	288959356
Rhodospirillum rubrum	RRU	83595063
Acidiphilium cryptum	ACR	148260328
Stigmatella aurantiaca	SUR	310819177
Desulfovibrio vulgaris	DVU	46580103
Anaeromyxobacter sp.	AFW	153003194
Anaeromyxobacter dehalogenans	ADE	86156723
Acidobacterium capsulatum	ACA	225873430
Candidatus Koribacter versatilis	ABA	94970054
Jannaschia sp.	JAN	89054991
Desulfurivibrio alkaliphilus	DAK	297569948

Sorangium cellulosum	SCL	162448842
Asticcacaulis excentricus	AEX	315498411
Brevundimonas subvibrioides	BSB	302382209
Phenylobacterium zucineum	PZU	197106301
Caulobacter sp.	CAK	167647167
Caulobacter crescentus	CCR	16126444
Caulobacter segnis	CSE	295690229
Parvibaculum lavamentivorans	PLA	154252083
Oligotropha carboxidovorans	OCA	209883896
Bradyrhizobium diazoefficiens	BJA	27376483
Nitrobacter hamburgensis	NHA	92118946
Rhodopseudomonas palustris	RPD	91975405
Xanthobacter autotrophicus	XAU	154247763
Azorhizobium caulinodans	AZC	158421996

Supplementary Table S3. NCBI-GI codes of 37 archaeal GluRS used in this work.

Name of species	KEGG code	NCBI-GI
Methanothermobacter thermautotrophicus	MTH	15678080
Methanobrevibacter smithii	MSI	148643512
Methanopyrus kandleri	MKA	20094194
Methanococcus maripaludis	MMP	45358574
Methanocaldococcus jannaschii	MJA	15669567
Nitrosopumilus maritimus	NMR	161527819
Cenarchaeum symbiosum	CSY	118576798
Methanohalobium evestigatum	MEV	298674188
Methanosarcina acetivorans	MAC	20089476
Archaeoglobus fulgidus	AFU	11497876
Ferroglobus placidus	FPL	288931867
Ignicoccus hospitalis	IHO	156937898
Thermococcus onnurineus	TON	212223335
Pyrococcus horikoshii	PHO	14591450
Natronomonas pharaonis	NPH	76802483
Halogeometricum borinquense	HBO	313125958
Natrialba magadii	NMG	289580129
Halalkalicoccus jeotgali	HJE	300711099
Halorhabdus utahensis	HUT	257053138
Haloarcula marismortui	HMA	55377019
Halobacterium sp.	HAL	15790230
Haloquadratum walsbyi	HWA	110668785
Nanoarchaeum equitans	NEQ	41615091
Thermofilum pendens	TPE	119719725
Methanohalophilus mahii	MMH	294496117
Methanocorpusculum labreanum	MLA	124485569
Picrophilus torridus	PTO	48477950
Aciduliprofundum boonei	ABI	289595769
Caldivirga maquilingensis	CMA	159042094
Hyperthermus butylicus	HBU	124027427
Acidilobus saccharovorans	ASC	302348133
Thermosphaera aggregans	TAG	296243110
Staphylothermus hellenicus	SHC	297527603
Desulfurococcus kamchatkensis	DKA	218884665
Sulfolobus acidocaldarius	SAI	70607261
Metallosphaera sedula	MSE	146304878
Sulfolobus islandicus	SIS	227831048

Supplementary Table S4. NCBI-GI codes of 22 eukaryal GluRS and 11 GluProRS used in this work.

	Name of species	KEGG code	NCBI-GI
**GluRS**	*Brugia malayi*	BMY	170572449
	Dictyostelium discoideum	DDI	66824081
	Bos taurus	BTA	529006739
	Danio rerio	DRE	113673058
	Aedes aegypti	AAG	157111006
	Acyrthosiphon pisum	API	328707259
	Schistosoma mansoni	SMM	256072189
	Aspergillus fumigatus	AFM	146323058
	Plasmodium yoelii	PYO	81177593
	Plasmodium vivax	PVX	156101325
	Plasmodium falciparum	PFA	124513854
	Babesia bovis	BBO	156087156
	Phaeodactylum tricornutum	PTI	219129351
	Arabidopsis thaliana	ATH	15237594
	Ricinus communis	RCU	255583454
	Trypanosoma brucei	TBR	72390563
	Leishmania major	LMA	157872813
	Lodderomyces elongisporus	LEL	149234782
	Saccharomyces cerevisiae	SCE	37362649
	Neurospora crassa	NCR	85091295
	Entamoeba histolytica	EHI	67480871
	Encephalitozoon cuniculi	ECU	19074040
**GluProRS**	*Equus caballus*	ECB	149743790
	Xenopus laevis	XTR	512844004
	Rattus norvegicus	RNO	66793366
	Mus musculus	MMU	82617575
	Pan troglodytes	PTR	114572699
	Canis familiaris	CFA	545556719
	Sus scrofa	SSC	545852051
	Gallus gallus	GGA	57530349
	Culex quinquefasciatus	CQU	170063637
	Drosophila melanogaster	DME	24649466
	Homo sapiens	HSA	62241042
